# Schizophrenia endothelial cells exhibit higher permeability and altered angiogenesis patterns in patient-derived organoids

**DOI:** 10.1038/s41398-024-02740-2

**Published:** 2024-01-23

**Authors:** Isidora Stankovic, Michael Notaras, Paul Wolujewicz, Tyler Lu, Raphael Lis, M. Elizabeth Ross, Dilek Colak

**Affiliations:** 1 Center for Neurogenetics, Feil Family Brain and Mind Research Institute, Weill Cornell Medicine, Cornell University, New York, NY USA; 2 Ansary Stem Cell Institute, Division of Regenerative Medicine, Department of Medicine, Weill Cornell Medicine, New York, NY USA; 3 Ronald O. Perelman and Claudia Cohen Center for Reproductive Medicine, Weill Cornell Medicine, New York, NY USA; 4 Gale and Ira Drukier Institute for Children’s Health, Weill Cornell Medicine, Cornell University, New York, NY USA

**Keywords:** Molecular neuroscience, Diseases

## Abstract

Schizophrenia (SCZ) is a complex neurodevelopmental disorder characterized by the manifestation of psychiatric symptoms in early adulthood. While many research avenues into the origins of SCZ during brain development have been explored, the contribution of endothelial/vascular dysfunction to the disease remains largely elusive. To model the neuropathology of SCZ during early critical periods of brain development, we utilized patient-derived induced pluripotent stem cells (iPSCs) to generate 3D cerebral organoids and define cell-specific signatures of disease. Single-cell RNA sequencing revealed that while SCZ organoids were similar in their macromolecular diversity to organoids generated from healthy controls (CTRL), SCZ organoids exhibited a higher percentage of endothelial cells when normalized to total cell numbers. Additionally, when compared to CTRL, differential gene expression analysis revealed a significant enrichment in genes that function in vessel formation, vascular regulation, and inflammatory response in SCZ endothelial cells. In line with these findings, data from 23 donors demonstrated that PECAM1^+^ microvascular vessel-like structures were increased in length and number in SCZ organoids in comparison to CTRL organoids. Furthermore, we report that patient-derived endothelial cells displayed higher paracellular permeability, implicating elevated vascular activity. Collectively, our data identified altered gene expression patterns, vessel-like structural changes, and enhanced permeability of endothelial cells in patient-derived models of SCZ. Hence, brain microvascular cells could play a role in the etiology of SCZ by modulating the permeability of the developing blood brain barrier (BBB).

## Introduction

SCZ is a complex psychiatric disorder that affects around 1% of the world population. It is characterized by a broad spectrum of symptoms, most prominent of which are delusions, hallucinations, behavioral and cognitive dysfunction [1, 2]. Despite the number of studies attempting to pinpoint causes of SCZ pathology [3–5] the initial insults, progression, and pathophysiology of this disease remain enigmatic and therefore effective treatment is lacking. In fact, approximately 30% of patients report little to no improvement with current pharmacological interventions [6]. While disease symptoms manifest in late adolescence and early adulthood, SCZ is largely regarded as a neurodevelopmental disease [7, 8]. Indeed, several lines of evidence point to intrauterine brain development as a key time point for disease onset given that multiple genetic and environmental factors converge here [4, 5, 9–11]. Epidemiological studies have described environmental variables such as prenatal stress as risk factors for SCZ onset. Concomitantly, patient-derived models of SCZ have identified genetic and proteomic alterations in the developing nervous system [12, 13]. Despite decades of research, identification of common pathways regulating ontogenesis of SCZ in the developing brain is severely limited by ethical and technical constraints surrounding the collection and usage of fetal brain tissue [13, 14]. Moreover, the majority of aforementioned studies focus on describing neuronal deficiencies, while a growing body of molecular evidence links the origins of SCZ to neuroinflammation, blood-brain barrier (BBB) dysfunction, vascular abnormalities and hypoxia in the developing brain [15–18].

Although alterations in brain vasculature and BBB permeability are linked to the onset of neurological disorders such as epilepsy, Alzheimer’s Disease, and traumatic brain injury [19–21] the contribution of these processes to SCZ pathology is largely elusive. Brain vascularization and BBB formation start early in embryogenesis with VEGF-A release mediating the recruitment of angioblasts and endothelial cells from the developing neural tube to generate the perineural vascular plexus (PNVP) [22, 23]. Once the PNVP is established, the BBB is formed through angiogenesis. The major unit of the BBB is the cerebral microvascular endothelium, which together with astrocytes, pericytes, microglia and neurons, constitute a neurovascular unit (NVU) [23]. NVU cells play an integral role in all stages of BBB development and maintenance and their function is key for transport of molecules (metabolites, nutrients, cytokines) and cells (immune cells, bacteria) to the brain [23, 24]. Of note, endothelial cells create a physical barrier to paracellular permeability by forming inter-endothelial tight junctions [25], while neurons have been shown to modulate local microcirculation and BBB permeability through release of various factors such as VEGF-A [26]. Moreover, neurovascular endothelial cells interact with surrounding astrocytes and other glial cells to regulate the efflux of immune factors and water-soluble molecules into the brain [27, 28].

Epidemiological data associates SCZ with cardiovascular disease, metabolic syndrome, and type 2 diabetes mellitus [29–31] conditions known to result from vascular endothelial dysfunction. To date, altered BBB permeability has been described in SCZ patients through clinical observations and post-mortem studies [32–35] indicating potential changes in NVU activity. However, most post-mortem studies only provide insight into the endpoint pathology of neuropsychiatric diseases. Thus, whether deficits in NVUs and BBB are primary pathologies of the disease, or a secondary/compensatory outcome is yet to be resolved. A recent study reported that SCZ patient-derived endothelial cells exhibit hyperpermeability compared to endothelial cells generated from CTRL subjects in 2D cultures [36] suggesting BBB deficits might be primary to the disease. However, the authors noted limitations of incomplete differentiation protocols leading to conflicting phenotypes of angiogenesis and permeability. Nevertheless, morphological characterization of NVUs and molecular profile of endothelial cells in early developing cortices of SCZ remain to be determined.

Previously, our lab utilized iPSCs to generate 3D cerebral organoids and model neuropathology of SCZ during critical periods of intrauterine brain development [12, 13]. In the present study, we employ patient-derived cerebral organoids to study NVUs and, for the first time, define molecular profiles of SCZ endothelial cells in 3D human-derived tissue. By combining a 3D cerebral organoid model with single-cell RNA sequencing (scRNA-seq) and immunohistochemistry, we found an increase in the number of brain microvascular endothelial cells, enrichment in the expression of NVU genes associated with BBB permeability, and hypervascularization-like structures in SCZ brain organoids. These novel findings were accompanied with hyper-paracellular permeability of SCZ endothelial cells generated from the same iPSC lines in 2D cultures. Together, our results have identified intrinsic structural and molecular alterations of brain endothelial cells within developing cortical fields of SCZ organoids. Our findings suggest that, in the developing brain, neurovascular endothelial dysfunction and BBB hyperpermeability could be contributing sources for the onset or progression of SCZ.

## Results

### Single-cell RNA sequencing reveals that cerebral organoids contain cellular components of the developing neurovascular unit

**Fig. 1:**
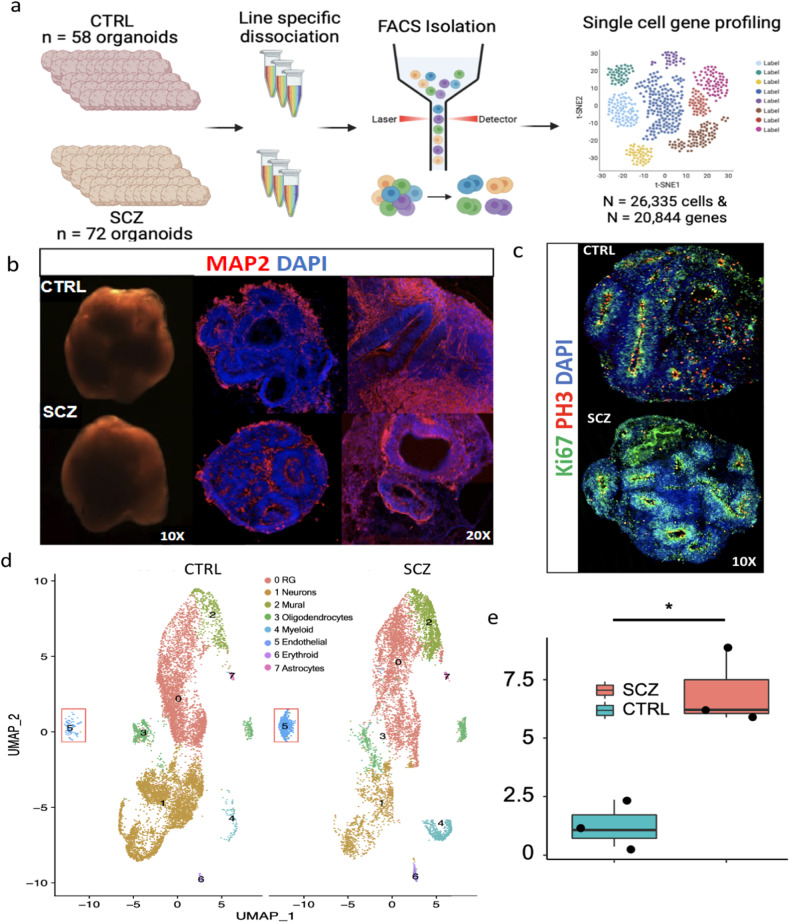
Single-cell RNA sequencing reveals that cerebral organoids recapitulate developing neurovascular unit. **a** Pipeline for performing scRNA-seq from brain organoids. Briefly, organoids from 3 CTRL and 3 SCZ lines were generated, pooled by line, and dissociated to a single-cell suspension. Samples were purified to 2000 live cells/µm per line and post-FACS cell viability was confirmed using Countess-II (See Supplementary Tables 1–3 for details about the donors and cell lines). Live cell suspensions were next rapidly loaded into 10x chromium microfluidic devices to produce barcoded single-cell nanodroplet emulsions. This emulsion was broken, barcoded samples were library prepared, Illumina sequenced, and analyzed. **b** Representative light microscopy images of 30 days in vitro (DIV) CTRL and SCZ organoids (left). Representative images of CTRL and SCZ organoids immunostained for neuronal marker MAP2 (red) and nuclear marker DAPI (blue). **c** Representative images of immunostainings for Ki67 (green) and PH3 (red) displays that CTRL and SCZ brain organoids recapitulate proliferative zones at 30 DIV. **d** UMAP coordinates for 26,335 transcriptomes split by CTRL and SCZ cases, presenting the cell-type clusters identified in unbiased clustering analysis. Unbiased gene sets were defined by the top 10 gene markers for each cluster that met a high-pass FDR threshold of 1% and >15,000 total read counts. Cell-type labels were determined via a variety of approaches including marker gene-expression, automated annotation, and comparison with human fetal samples (see “Materials and methods“ for further details). Endothelial cell clusters are denoted by a red square. **e** Bar graph depicting a significant difference in endothelial cell number (cluster 5) between CTRL and SCZ organoids. Each point on the graph represents an independent cell line. *p < 0.05. Error bars reflect Standard Error of the Mean.

To characterize neurovascular cells in developing SCZ cortical fields, we generated self-assembling, self-maturating, and human-derived cerebral organoids [13, 37, 38]. Cerebral organoids broadly mimic the first trimester of human brain development, a time point previously described as an early risk period for SCZ [39–41]. It has been shown that cerebral organoids recapitulate both transcriptomic and epigenomic programs of developing fetal brain, and thus have been previously used to study mechanisms of both brain development and disease [13, 38, 42–44]. To date, several labs have generated models of vascularized cerebral organoids, by engineering iPSCs to express vascular transcription factors such as ETV2 [45–48]. However, to retain disease-specific neurovascular deficits, we generated self-developing organoids with spontaneously formed microvasculature and unbiasedly profiled endothelial cells using scRNA-seq (Fig. 1a). Recent organoid models often apply exogenous growth factors (e.g., BDNF, NT-3, EGF) and/or pathway modulators (e.g., TGFβ and WNT pathway inhibitors) to direct the differentiation of progenitors towards a neuronal fate or promote their survival by reducing apoptosis. However, factors such as BDNF and related neurotrophins, TGFβ, and WNT signaling are longstanding contributors to SCZ pathophysiology and may play a role in the ontogenesis of disease during early neurodevelopment. Indeed, these same factors and pathways support hypotheses on early disease development via their effects on stem cells and neuronal programming. In fact, scRNA-seq analysis revealed that progenitors in SCZ organoids (generated via a morphogen-free protocol) exhibited down-regulated *BDNF* and *TrkB*, but up-regulated *NT-4*, gene-expression [13], pointing to the importance of organoid protocol selection to fully detect disease phenotypes. Therefore, we adapted a morphogen-free organoid protocol for our primary experiments to avoid these potential confounding factors. Consistent with our recently published studies [12, 13], CTRL and SCZ organoids did not differ in morphology or generation of neuronal and proliferative zones (Fig. 1b, c), as observed by MAP2 and Ki67/PH3 immunostainings, respectively. We have also established organoid reproducibility via computational analysis [13] and verified it with extensive measurements of proliferation, progenitor pools, neuronal induction, and cell death to confirm low baseline variability (Supplementary Fig. 1, see also ref. [13]). To study disease signatures of SCZ with greater resolution, both with respect to cell-specificity and defined molecular profile, we employed scRNA-seq. Specifically, this unbiased analysis resulted in 26,335 single-cell transcriptomes, comprising 20,844 captured genes that could be grouped and harmonized into 8 cellular clusters (identified against human fetal brain samples). SCZ organoids mirrored cell-types present in CTRL organoids (Fig. 1d). Moreover, both CTRL and SCZ organoids contained cells comprising early NVUs including neurons, radial-glial cells, oligodendrocytes, endothelial cells, erythroids and astrocytes (Fig. 1d). Compared to CTRL organoids, SCZ organoids were defined by a decrease in neuron numbers (Fig. 1d), a phenotype extensively described in our recent publication [13]. Intriguingly, unbiased scRNA-seq analysis revealed an increase in other neurovascular cell types, with a significant increase in endothelial cells (Fig. 1e). See also “Methods” section and Supplementary Tables 1–3 for information about the CTRL and SCZ iPSC lines including clinical phenotypes as well as specific cell lines used for each experiment.

### Endothelial cells and microvascular vessel-like structures are increased in SCZ brain organoids

**Fig. 2:**
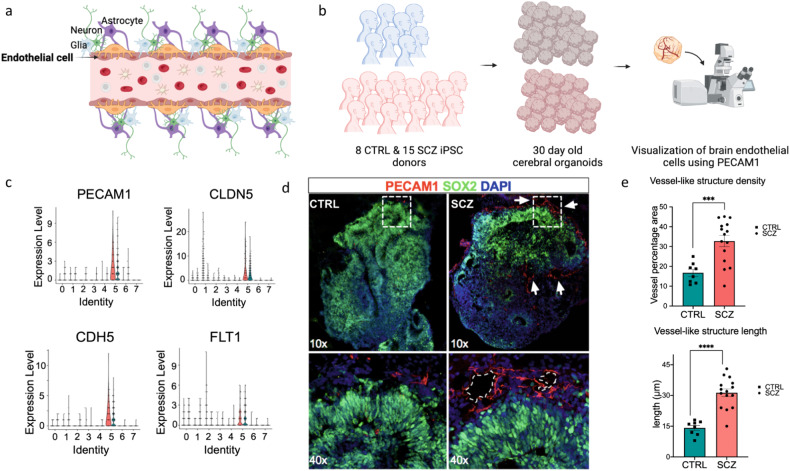
Endothelial cells and microvascular vessel-like structures are increased in SCZ brain organoids. **a** Schematic of neurovascular unit comprising various brain cell types. Endothelial cells, which are highlighted, line the blood vessels, and interact with the surrounding cells. **b** Schematic of the pipeline to image endothelial cells and microvascular vessel-like structures in organoids. In total, *n* = 23 distinct iPSC lines (8 CTRL, 15 SCZ) were used to generate organoids to visualize and quantify endothelial cells. **c** Violin plots depicting gene expression profiles for known endothelial genes across each cell population cluster and by conditions: PECAM1 (adhesion), CLDN5 (tight junction regulation), CDH5 (proliferation of ECs), FLT1 (tight junction regulation), plotted for each of the seven clusters. **d** Representative images of PECAM1 immunostainings in CTRL and SCZ organoids depicting microvascular vessel-like structures. Neural progenitors were labeled using SOX2 antibody and nuclei using DAPI. Squares and arrows highlight microvascular vessel-like structures in both conditions. **e** Quantifications revealed significant differences in the lengths as well as densities of vessel-like structures between CTRL and SCZ organoids. Each point in the bar graphs represent an independent line. For the measurements of density, briefly, raw images were 16 bit converted and skeletonized, and vessels were counted and normalized per organoid area. A minimum of 5 organoids were counted per line. For the measurements of density, briefly, raw images were 16 bit converted and skeletonized - a vessel like structure was counted as a singular branch longer than 1 µm (see Supplementary Fig. 2 for data split by line). ***p < 0.001 and ****p < 0.0001. Error bars reflect Standard Error of the Mean.

In the NVU, endothelial cells make up the walls of the blood vessels, tightly controlling their permeability (Fig. 2a) [17, 49]. To histologically validate the scRNA-seq phenotype of SCZ endothelial cell density, we first quantified endothelial cells within cortical fields of SCZ and CTRL organoids using immunohistochemistry. To do this, we employed organoids generated from 23 independent iPSC lines (8 CTRL, 15 SCZ), grown for 30 *days* in vitro (the same time point scRNA-seq was performed) (Fig. 2b). The scRNA-seq analysis revealed higher expression of common endothelial markers including, adhesion markers (PECAM1, CDH5), tight junction marker CLDN5, and growth factor receptor FLT1 in SCZ brain organoids (Fig. 2c). PECAM1 immunostaining confirmed the presence of PECAM1-expressing cells (PECAM1^+^) both in CTRL and SCZ organoids (Fig. 2d). Quantification of the signal validated the scRNA-seq finding that there is a significant increase in total PECAM1 signal in SCZ organoids compared to CTRL organoids (Fig. 2e). It was reported that PECAM1^+^ endothelial cells were capable of self-organizing into vessel-like structures within brain organoids [50, 51]. Thus, we also took advantage of the 3D tissue to assess morphology and density of microvascular-like structures using PECAM1 signal. By doing so, we discovered that the total number of independent vessel-like structures were increased in SCZ organoids compared to CTRL organoids (Fig. 2e). However, split analysis by iPSC lines revealed that there were four outliers among the 15 SCZ lines quantified (Supplementary Fig. 2a). PECAM1^+^ endothelial vessel-like structures also formed interconnected networks both in CTRL and SCZ organoids (see arrows in upper panels of Fig. 2d). We next sought to compare the length of those interconnected networks between SCZ and CTRL organoids. Similar to the density phenotype, we found that the lengths of the vessel-like structures were also increased in SCZ organoids compared to CTRL (Fig. 2e). For this phenotype only one line was an outlier among the 15 lines (Supplementary Fig. 2b). Together, these suggest that increase in PECAM1 signal intensity in SCZ organoids does not solely arise from higher expression within a given unit, but rather is a result of increased number and length of PECAM1^+^ vessel-like structures in patient-derived cerebral tissue.

### Endothelial cells of SCZ patient-derived brain organoids exhibit changes in angiogenic pathways and cell cycle regulation

**Fig. 3:**
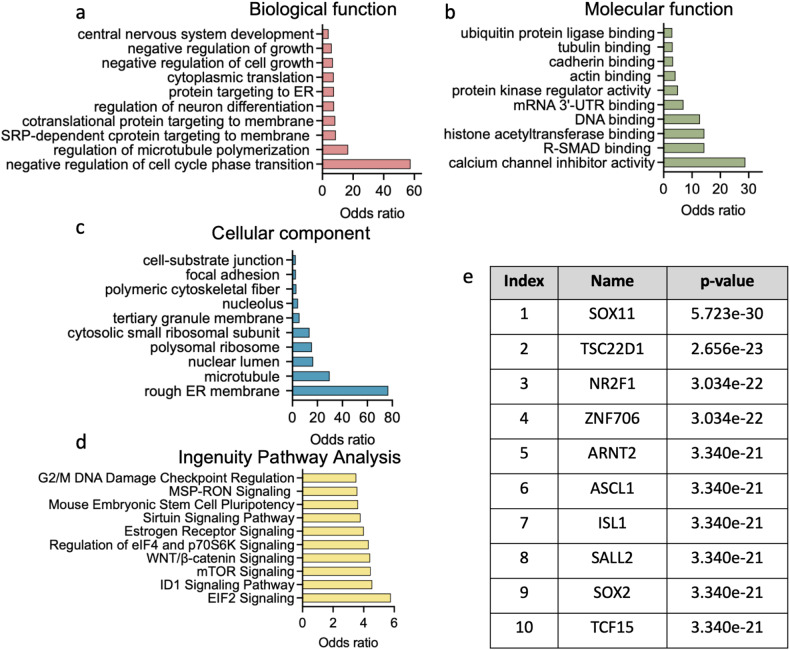
Endothelial cells of SCZ patient-derived brain organoids exhibit changes in angiogenic pathways and cell cycle regulation. **a** Gene Ontology (GO) analysis for biological function displaying the top categories represented differentially expressed genes (DEGs) of SCZ endothelial cells; odds ratio as observed between SCZ and CTRLs (see Supplementary Table 4 for the list of DEGs). **b** GO analysis for molecular function displaying the top categories represented in SCZ endothelial DEGs. **c** GO analysis for cellular components displaying the top categories differentially expressed in SCZ endothelial DEGs. **d** Ingenuity Pathway Analysis displaying the top most represented signaling pathways in SCZ endothelial cells compared to CTRL. **e** Top transcription factors listed with significance *p*-values predicted to co-occur with SCZ endothelial DEGs.

We next sought to bioinformatically profile and compare gene expression patterns in endothelial clusters of CTRL and SCZ organoids. In total, 260 genes were found to be differentially expressed in SCZ endothelial cluster compared to CTRL endothelial cluster (Supplementary Table 4). Intriguingly, only 30 genes were upregulated while 230 genes were downregulated in SCZ organoids compared to CTRL. We first grouped the differentially regulated genes based on their biological function, molecular function, cellular component, and pathway represented (Fig. 3a–d). The most significantly altered biological functions were cell cycle regulation and microtubule polymerization. Molecular function analysis determined that the most significantly altered process was calcium channel inhibitor activity (Fig. 3b). Intriguingly, calcium channel inhibitors modulate perfusion by allowing blood vessels to relax and open [52–54]. Among the 260 differentially regulated genes, microtubule and ER associated genes compromised the most enriched cellular components (Fig. 3c). Significantly altered pathways included diverse types of cascades that are linked to endothelial cell generation or function such as ElF2, ID1, and mTOR signaling. EIF2 signaling is comprised of several endothelial cell transcription factors and regulates both cell cycle progression and angiogenesis [55–57], while ID1 signaling activates HIF-1a and VEGF-A to promote tumor angiogenesis in certain cancers [58]. Concurrently, the mTOR pathway in endothelial cells has been shown to regulate angiogenic sprouting, migration, cytoskeleton reorganization, and signaling events impacting matrix adhesion [59, 60].

Among the differentially expressed genes, several transcription factors were detected (Fig. 3e). The most significantly downregulated transcription factor in SCZ endothelial cells compared to CTRL cells was SOX11. On the contrary, SOX2 from the same family was upregulated in SCZ endothelial cells. SOX transcription factors play an important role in vascular development and disease [61]. Specifically, SOX2 is not only required for endothelial cell differentiation but can also induce endothelial differentiation in isolated mesoangioblasts, which are mesenchymal-like cells associated with the walls of vessels [62]. SOX11 was also reported to promote tumor angiogenesis in certain forms of lymphoma [63]. Among the other differentially regulated transcription factors (TF), TSC22D1 is a known marker of peripheral endothelial cells and was previously identified as a BBB enriched gene [64], while ARNT was shown to regulate blood vessel dilation [65].

Taken together, genes differentially expressed in endothelial cells of SCZ organoids were reported to function in angiogenesis, regulation of vasculature, and fine-tuning cell cycle progression.

### Endothelial cells in SCZ brain organoids exhibit transcriptional changes linked to CD40 regulation

**Fig. 4:**
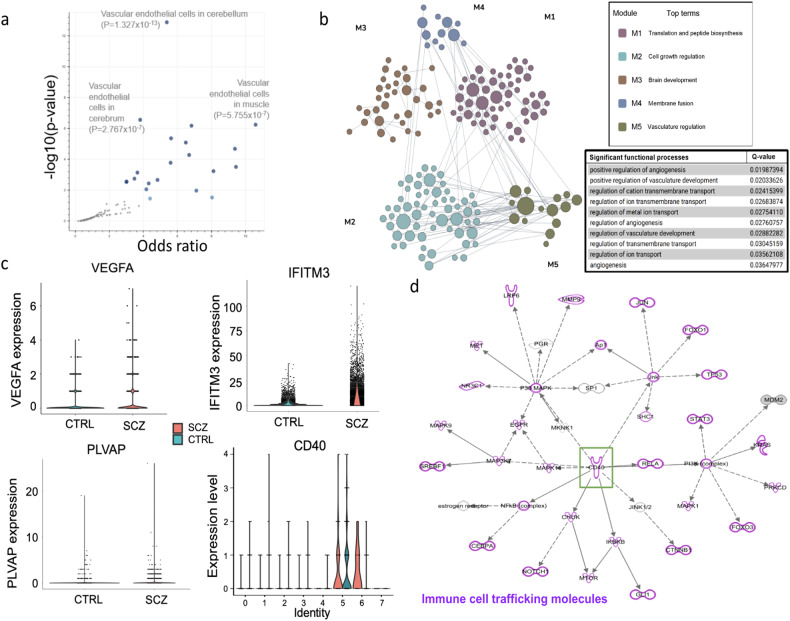
Endothelial cells in SCZ brain organoids exhibit transcriptional changes linked to CD40 regulation. **a** Volcano plot of terms from the DESCARTES Cell types and Tissue 2021 library gene set. Each point represents a single term, plotted by the corresponding odds ratio (x-position) and −log10(*p*-value) (y-position) from the enrichment results of the input query gene set. The larger and darker-colored the point, the more significantly enriched the input gene set is for the term. **b** Brain-specific functional module prediction for DEGs detected in the endothelial population of SCZ samples. The significant functional processes in module 5 related to vasculature regulation are highlighted along with their corresponding Q-values. **c** Violin plot of VEGF-A, IFITM3, PLVAP expressions in cluster 5 of scRNA-seq showing differences in expression in CTRL and SCZ. Violin plot for CD40 showing gene expression in cell clusters 0-7 (x-position) in both CTRL and SCZ samples. **d** IPA-Causal networks analysis predicted CD40 as a top regulator amongst the endothelial population enrichment in SCZ samples. Green square highlights CD40 in the center of causal network analysis. Purple highlights immune cells trafficking molecules known to interact with CD40.

We next sought to determine if there is a common upstream mechanism underlying the differentially regulated gene expression pattern between SCZ and CTRL endothelial cells. As expected, when the differentially regulated genes were grouped into neurologically relevant pathways, genes comprising human endothelial cells, embryonic vasculature, and pericytes contained the highest enrichment (Fig. 4a). The differentially expressed genes composed 5 distinct interaction modules (Fig. 4b). One of these gene modules, Module 5, included genes that function in vasculature regulation, angiogenesis, and vascular development.

Because VEGF-A acts as a key angiogenic stimulus for new blood vessel growth in the developing brain [66–68], we specifically examined expression of VEGF-A in the CTRL and SCZ endothelial clusters. Intriguingly, VEGF-A transcript was increased in SCZ endothelial cells compared to CTRL endothelial cells (Fig. 4c). Of note, in addition to regulating angiogenesis, VEGF-A has also been suggested as a regulator of permeability in both normal and disease states by increasing microvascular permeability to blood plasma proteins within minutes of exposure [69, 70]. Given that only 30 genes of 260 differentially expressed genes were upregulated in SCZ organoids, we further examined whether any are related to endothelial dysfunction. Among the most highly enriched were two upregulated genes previously implicated in neuroinflammatory phenotypes in SCZ brains: interferon induced transmembrane protein 3 (IFITM3) and plasmalemma vesicle-associated protein (PLVAP) (Fig. 4c). Inflammatory factor IFITM3 enrichment was previously shown in the hippocampus, amygdala and the frontal cortex of postmortem SCZ brains [71–73] and thought to reflect increased neuroinflammation. The function of endothelial-cell specific PLVAP is to form the diaphragm between endothelial fenestrae and regulate permeability, immune cell migration, and angiogenesis [74]. Similar to IFITM3 upregulation in postmortem SCZ brains, greater levels of endothelial-cell specific PLVAP have been observed in peripheral blood of SCZ patients compared to healthy CTRLs [75]. Elevated levels of PLVAP leads to increased transcellular permeability of endothelial cells that in turn aggravates inflammation by increasing leukocyte trafficking [74].

Next, we mapped the gene interactions and identified the causal networks of the differentially regulated genes in SCZ endothelial cells via Ingenuity Pathway Analysis (IPA). The Causal Network Analysis within IPA exposes causal relationships associated with the data by expanding upstream analysis to include regulators that are not directly connected to targets in the given dataset. Our analysis predicted that the differentially expressed genes were commonly linked to the proinflammatory factor CD40 (Fig. 4d). CD40 is a membrane glycoprotein of the tumor necrosis factor receptor superfamily. It is expressed on most cell populations including lymphocytes, macrophages, platelets, endothelial, and neuronal cells. CD40 binds its ligand CD40L through the induction of cellular adhesion molecules of endothelial cells, and this CD40/CD40L dyad was previously shown to modulate the permeability of vessels in conditions such as HIV and inflammation [76, 77]. Of note, the CD40/CD40L dyad was shown to exacerbate endothelial cell dysfunction and activation by increasing oxidative stress and systemic inflammation [78]. While CD40 was not specifically differentially expressed in SCZ endothelial cells compared to CTRL cells, there was an overall upregulation in CD40 levels in SCZ organoids (most likely in several major cell types) compared to CTRL organoids (Fig. 4c), suggesting that SCZ organoids exist in a proinflammatory state.

### SCZ patient-derived endothelial cells exhibit increased paracellular permeability

**Fig. 5:**
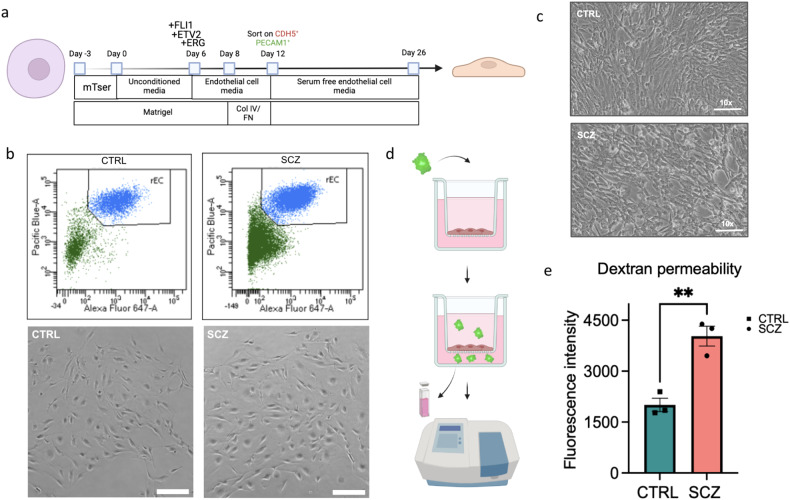
SCZ patient-derived endothelial cells exhibit increased permeability. **a** Schematic of induced brain microvascular endothelial cell (iBMEC) differentiation from iPSCs (See Supplementary Tables 2 and 3 for details about the donors and cell lines used in each experiment). For further details on differentiation see “Materials and methods”. **b** On day 12 of differentiation cells are labeled for CDH5, PECAM1 and sorted using flow cytometry to isolate a pure population of iBMECs. Representative flow cytometry plots of CTRL and SCZ iBMECs sorted for PECAM1 and CDH5 (top), and light microscopy images of CTRL and SCZ iBMECs after sorting (bottom, scale bar = 200 µm). **c** Light microscopy images of CTRL and SCZ iBMECs after day 26 of differentiation at ×10 magnification, preceding dextran permeability assay. **d** Schematic depicting the workflow of FITC-dextran assay to measure paracellular permeability of cultured iBMECs. FITC-dextran was added to the Transwells for 6 h. At the end of 6 h of treatment, the fluorescence intensity of the medium in the lower compartments was measured to assess paracellular permeability. Because the FITC-dextran is 70 kDA, it cannot get passively transported through the cells but can only pass through the Transwells if intercellular junction permeability is altered. **e** Graph depicting quantifications of FITC-dextran fluorescence permeability assay. Each dot represents a distinct donor line used in the experiment, *n* = 3 lines per group (See Supplementary Fig. 2c for data split analysis by line), **p < 0.01. Error bars reflect Standard Error of the Mean.

Paracellular transport involves the transfer of substances from the blood across the endothelium via intercellular spaces, and its tight regulation is the core property of brain microvascular endothelial cells. Because altered levels of various diverse types of transcripts that are differentially regulated in SCZ endothelial cells are linked to permeability, we next assessed paracellular permeability of CTRL and SCZ iPSC-derived endothelial cells in vitro. To do this, we generated bona fide induced brain microvascular endothelial cells (iBMEC) from CTRL and SCZ iPSCs using the most recent published protocol (Fig. 5a) [79]. Unlike previous protocols that generates a surplus of epithelial tissue rather than individual endothelial cells [36], the protocol we employed generates iBMECs from iPSCs through overexpression of key endothelial ETS family transcription factors ETV2, ERG, and FLI1 [79]. Upon induction of iPSC differentiation by ETV2, EFG, FLI1 overexpression, CTRL or SCZ cells were sorted based on CDH5, PECAM1 to isolate a pure population of endothelial cells (please see Materials and methods). Both CTRL and SCZ iPSCs successfully yielded pure BMECs (Fig. 5b) that proliferated and generated confluent cultures **(**Fig. 5c**)** (See also Supplementary Tables 1-3 for details about the donors and cell lines for this experiment).

To assess paracellular permeability in SCZ endothelial cells, we performed the well-established fluorescein isothiocyanate (FITC)-Dextran assay (Fig. 5d), which is typically used to evaluate the paracellular permeability of semi-permeable membranes in vitro [80]. This assay measures the fluorescence intensity of 70 kDa FITC-conjugated dextran permeating through monolayer-cultured cells and is commonly applied to analyze hyper- or hypo paracellular permeability states of endothelial cells [81]. We cultured 100,000 CTRL or SCZ iBMECs as a monolayer onto Transwell plates with 0.4-μm pore sized inserts. Once cells reached 100% confluency, FITC-dextran was added to the Transwells for 6 h. Because the FITC-dextran is 70 kDA, it cannot passively transport through cells but can only pass through the transwell if intercellular junction permeability is altered. At the end of 6 h of treatment, the fluorescence intensity of the medium in the lower compartments was measured by the BioTek Epoch Microplate Spectrophotometer at an excitation of 485 nm and emission of 530 nm. The FITC-conjugated dextran passage was calculated with a standard curve. The quantifications revealed that SCZ iBMECs permitted the passage of more FITC-dextran compared to CTRL cells (Fig. 5e). Unlike the PECAM1 quantifications (Fig. 1), there were no outliers in both CTRL and SCZ groups as cell lines across the given group exhibited minimal variability for the permeability phenotype (Supplementary Fig. 2c). This suggests that SCZ iBMECs exhibit a hyper-permeability phenotype.

## Discussion

**Fig. 6:**
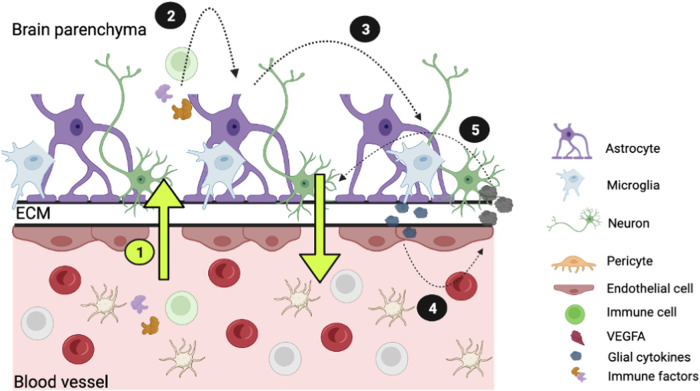
Proposed model of altered paracellular permeability of BMECs and the downstream inflammation response in SCZ. **1** Genetic factors contribute to endothelial junction changes resulting in altered paracellular permeability that allows proinflammatory cytokines to traverse into the parenchyma. Neon green highlight denotes that this is the part of the pathway in which we observe the most changes in our SCZ organoid and 2D iPSC systems. **2** Once in the parenchyma these inflammatory molecules activate astrocytes and microglia. **3** Astrocytic and microglial activation results in the release of additional proinflammatory cytokines that can traverse the vessels and parenchyma borders. **4** These proinflammatory cytokines will result in a feedback-loop activation of BMECs which will release BMEC specific cytokines. **5** Cytokines released by BMECs further recruit other cells (neutrophils, T cells, B cells) and alter adhesion molecules that contribute to vessel permeability seen in SCZ. The current study has specifically focused on endothelial cell alterations that could possibly contribute to the BBB/NVU pathology hypothesis of SCZ. Brain organoid protocols that recapitulate pericytes and microglia are yet to be developed. 2D cultures of induced BMECs lack the complexity of the multicellular nature of the BBB. Our initial characterization of SCZ iBMEC phenotypes should be expanded to characterize SCZ BBB dysfunction using the 3D models of BBB such as human BBB-Chip with iPSC-derived BMECs, iPSC-derived astrocytes, and iPSC-derived neurons.

Our study identifies endothelial cell alterations in SCZ patient organoids and supports the hypotheses that SCZ has early developmental origins associated with BBB dysfunction and proinflammatory states (Fig. 6). To date, SCZ remains a debilitating mental disorder whose etiology remains elusive. While many studies have explored the abnormalities in neurons and defects in cortical inhibition to the onset of SCZ [4, 5, 9–12], the role of other cell types have been underexplored. Post-mortem studies, genome-wide association and gene expression analysis have previously implicated BBB dysfunction in the onset of SCZ. However, the exact contribution of each cell type in the BBB remains elusive. Additional challenges in deciphering BBB dysfunction in SCZ is the timeline for when SCZ-related pathology initiates. While multiple theories have been put forth regarding the origin of SCZ, the evidence points to a neurodevelopmental model in which changes occur in early pregnancy (1-2 trimester) [7, 8], leading to pathological neural circuit function that displays symptoms in adulthood.

The BMECs that line blood vessels and interact with surrounding cells are a central element of the microvasculature that forms the BBB. To assess intrinsic BMEC alterations during brain development prior to SCZ symptoms, we have employed patient-derived cerebral organoids. Using this approach, we have previously defined neural-specific pathology in 3D human derived tissue corresponding to early developmental stages [12, 13]. Here, we have focused on the pathology of endothelial cells in SCZ brain organoids. Of note, we adapted a mmorphogen-free organoid system that takes advantage of the self-organizing capacity of stem cells and allows spontaneous generation of all cell types. The organoids were not treated with growth factors or factors that induce angiogenesis or vascularization that may interfere with disease specific pathways, thus likely preserving disease-specific features in endothelial cells. Using both organoids and induced BMECs we identified three major phenotypes in SCZ endothelial cells - altered cell numbers, increased angiogenesis, and elevated paracellular permeability.

ScRNA-seq is a powerful method to unbiasedly identify molecularly distinct cell types in heterogeneous tissues. How many cell clusters can be deciphered from scRNA-seq is largely dependent on the person annotating the analysis and the computational pipelines used. However, current literature including our work suggests that around 8-9 major distinct cell types can be identified within cerebral organoids. Although batch to batch analysis was not performed by us or others, in an independent study, the Treutlein lab identified a single cell type expressing PECAM1 and many other endothelial markers in organoids derived using the same protocol as ours [82] suggesting that recapitulation of endothelial cells is not an artifact in our organoids. ScRNA-seq of CTRL and SCZ organoids revealed that differentiation trajectories shifted from neurogenesis toward mural, myeloid, and endothelial cell lineages in patient organoids (Fig. 1d) (see also ref. [13]). Consistent with this, vascular-related metabolic processes and angiogenic markers of cerebrum were enriched in SCZ organoids (Fig. 2c). These markers included PECAM1, CLDN5, CDH5 and FLT1 (Fig. 2c). PECAM1 and CLDN5 are adhesion molecules known to regulate endothelial junction integrity [83, 84]. CDH5 and FLT1 regulate permeability of the BBB by regulating tight junctions and angiogenesis, respectively [85, 86]. Our histology analyses using organoids derived from 15 distinct SCZ donors confirmed that PECAM^+^ cells were increased in SCZ organoids in comparison to CTRL organoids (Fig. 2d; see also Fig. S2 for data split analysis). PECAM^+^ cells formed microvascular vessel-like structures within organoids that are comparable to those in induced vascularized organoid models [46, 47, 50]. Additionally, these cells generated more and longer vessel-like structures in SCZ organoids compared to CTRL (Fig. 2e). Evaluating morphology of blood vessels in postmortem SCZ brains is confounded by the implementation of atypical antipsychotics that affect cardiovascular function [87, 88], therefore the scope of research findings is limited. Nevertheless, some studies reported alterations in brain vessels [5] and changes of local vascular network structures [89, 90] in SCZ tissues. Meanwhile, several studies have reported elevated pro-inflammatory cytokines in both blood and brains of individuals with SCZ, implicating altered BBB permeability in the disease [35, 91, 92]. Our results demonstrate, for the first time, that there is an early-developmental increase in endothelial cell numbers in a 3D brain tissue model of SCZ in vitro.

Here, we show that alterations in vascular and angiogenesis gene expression are intrinsic to developing SCZ tissue, and thus may not be secondary to the disease. SCZ is associated with chronic low-grade inflammation, which has been linked to increased vascular risk [93, 94]. Studies have reported higher VEGF levels in individuals with SCZ compared to healthy CTRLs [95]. During embryonic brain development, angiogenesis, the sprouting of new blood vessels, is spatially and temporally controlled by expression of VEGF-A by neurons and glia [25]. After angiogenesis starts, BMECs show diminished leakage and a higher expression of tight junction proteins, such as CLN5 and OCLN [23, 25]. In SCZ organoids, genes and regulators that function in angiogenesis and vascularization are significantly increased compared to CTRL organoids (Fig. 4a, b). Intriguingly, regulation of microtubules, which are required to stabilize endothelial cell protrusions [96], is one of the most affected biological functions in SCZ endothelial cells (Fig. 3a). R-SMAD regulation is crucial for sprouting of new blood vessels as the Smad 2/3-Alk cascade is indispensable for vascular stability and N-cadherin expression [97]. Our molecular function analysis indicated R-SMAD binding and calcium channel inhibitor activity are the most highly differential in SCZ endothelial cells, and their alterations could also contribute to vessel-like phenotypes in SCZ organoids (Fig. 3 a–c). The endothelium plays a significant role in modulating vascular tone and cerebral blood flow via calcium channels, electrical signaling, and endothelial gap junctions [98–100]. Calcium is an essential second messenger in endothelial cells and plays a pivotal role in regulating a number of physiologic processes, including cell migration, angiogenesis, barrier function, and inflammation [101]. Additionally, pathway analysis revealed that ElD2, ID1, and mTOR signaling, which have all been implicated in angiogenesis [102–104], were amongst the most significantly affected pathways in SCZ endothelial cells compared to CTRL endothelial cells. Moreover, the VEGF-A, the most prominent angiogenic factor of endothelial cells [105, 106], was significantly upregulated in SCZ endothelial cells compared to CTRL cells **(**Fig. 4c**)**. Intriguingly, ID1 signaling was recently shown to activate VEGF-A to promote angiogenesis [58]. Taken together, in our organoid system there is substantial evidence of increased angiogenic potential in SCZ compared to CTRL, a possible effect of enriched ID1, ElF2 and mTOR pathways.

Our data supports the hypothesis that inflammation may be an intrinsic component of early developmental neuropathology in SCZ patient-derived organoids. We found that the inflammatory factor IFITM3 was upregulated in SCZ endothelial cells compared to CTRL endothelial cells in organoids (Fig. 4c). IFITM3 has been suggested to mediate perinatal/neonatal immune activation effects on the brain [107] and, consistent with this, knockout mice are protected from neuronal pathology induced by models of maternal immune activation [108]. Moreover, IFITM3 has been identified as a novel drug target candidate in hyperinflammatory SCZ [109], as it was reported to be enriched in the hippocampus, amygdala and the frontal cortex of post-mortem SCZ brains [71–73]. In addition to IFITM3, the plasmalemma vesicle-associated protein PLVAP, which is commonly considered to be endothelium-specific, was also upregulated in endothelial cells of SCZ organoids compared to CTRL samples. PLVAP forms the diaphragm that bridges endothelial fenestrae and regulates permeability, leukocyte transmigration, and angiogenesis [17, 74]. Increased levels of PLVAP have been observed in the peripheral blood of SCZ patients in comparison to non-deficit SCZ or CTRLs [75]. Like IFITM3, PLVAP is also associated with inflammation and, in fact, is suggested to aggravate inflammation by increasing leukocyte trafficking [74]. Our findings suggest that neuroinflammation may be an intrinsic SCZ phenotype during early brain development. In fact, causal network analysis predicted proinflammatory factor CD40 as a likely common link to differentially regulated genes in SCZ endothelial cells (Fig. 4d). Intriguingly, CD40 levels were also upregulated in SCZ organoids compared to CTRL organoids (Fig. 4c), supporting the hypothesis that inflammation may be an intrinsic component of early developmental neuropathology in SCZ.

We report that paracellular permeability of patient SCZ iPSC-derived brain endothelial cells (iBMECs) is increased compared to CTRL iBMECs in vitro (Fig. 5a–e). Due to low-grade inflammation consistently observed in postmortem brains of SCZ patients, one hypothesis is that SCZ is a disease of altered BBB permeability [110–112]. However, whether inflammation is primary or secondary to the disease remains unknown. Similarly, BBB permeability deficits have not been well established in SCZ cases or models. Nevertheless, increased CSF:serum albumin ratio in patients with SCZ [113] as well as observation of lymphocytes in various regions of postmortem SCZ brains [114] suggest that increased BBB permeability is associated with the disease. Permeability of the BBB is dependent on tight cell-cell contacts mediated by tight junction complexes, low levels of paracellular diffusion, and low transcellular endocytosis. Our unbiased scRNA-seq data revealed altered pathways and direct regulators of permeability as well as increased levels of inflammation factors in SCZ endothelial cells (Fig. 3b). Systemic inflammation is known to increase permeability of BBB [109, 110]. In addition to increased levels of proinflammatory CD40 in SCZ organoids, upregulation of neuroinflammatory factor IFITM3 and transcellular permeability regulator PLVAP in SCZ endothelial cells within brain organoids implicate permeability deficits in endothelial cells in 3D brain tissue model of SCZ. Our in vitro paracellular permeability assay has confirmed higher permeability in SCZ iBMECs supporting the hypothesis that BBB dysfunction might have developmental origins in SCZ (Fig. 5e).

In conclusion, our study identified specific endothelial cell alterations in patient derived SCZ tissue. While hypotheses that SCZ might be a disease of altered blood vessels have been discussed for over 100 years [115–117], evidence of alterations during critical periods of brain development has been largely missing. Without a way to ethically acquire, study, and subsequently identify whether human fetal brain would definitively become a future SCZ case, little progress has been made in understanding early developmental mechanisms of the disease. By using a combination of 3D brain organoids that recapitulate fetal brain development and 2D bona-fide induced brain endothelial cells, this study identifies the early origins of SCZ endothelial alterations and possible BBB dysfunction. Thus, we suggest that neuropathology of SCZ is likely to be substantially more nuanced than currently accepted, with dysfunction of the vascular-endothelial network during brain development playing an important role in the onset of SCZ (Fig. 6).

### Limitations and future directions

Due to sparse distribution of endothelial cells within organoids, we were unable to isolate these cells from organoids and propagate them to test permeability. While direct differentiation strategy allowed us to test permeability of SCZ endothelial cells, whether the molecular profiles and functional capacities of iBMECs in vitro and endothelial cells within brain organoids are comparable is not clear. A core property of BMECs is the strict regulation of paracellular permeability. Paracellular transport involves the transfer of substances from the blood across the endothelium via intercellular spaces (Fig. 6). This process is controlled by a complex arrangement of tight, gap, and adherens junctions that contribute to tissue integrity, barrier function, and cell-to-cell communication. Thus, here, we prioritized and studied the paracellular permeability of SCZ iBMECs. However, BMECs also possess a high degree of trans-endothelial electrical resistance (TEER), which indicates barrier integrity. During inflammation trans-endothelial permeability allows for attachment and transmigration of leukocytes across the BBB. In future studies, assessment of TEER will be valuable to provide further insight into the pathophysiology of SCZ iBMECs.

ScRNA-seq is a powerful method to unbiasedly identify molecularly distinct cell types in heterogeneous tissues. Here, it allowed us to identify alterations specific to the endothelial cell population of SCZ brain organoids. Although transcriptomics provides a useful overview of gene expression, it cannot capture posttranslational modifications. Thus, proteome analysis is required to gain comprehensive insight into the protein profile of CTRL or SCZ endothelial cells. Because current proteomics methods cannot capture cell specificity in heterogeneous tissues, combination of cell surface labeling or reporter constructs with fluorescence-activated cell sorting would allow to assess protein alterations in endothelial cells of SCZ organoids.

Homogeneous population of patient iPSC-derived BMECs recapitulate certain aspects of endothelial functions and allow identification of molecular and cellular deficits that might contribute to SCZ. However, 2D cultures of induced BMECs lack the complexity of the multicellular nature of the BBB. 3D BBB models are being developed to recapitulate direct interaction between BMECs and other NVU cells and thereby enhance endothelial cell maturation. A recent study successfully created human BBB-Chip with iPSC-derived BMECs, iPSC-derived astrocytes, and iPSC-derived neurons [118]. In future studies, our initial characterization of SCZ iBMEC phenotypes could be expanded to characterize SCZ BBB dysfunction using the 3D models of BBB.

Another promising research direction could be focusing on the contribution of microtubule regulation in endothelial cell dysfunction in patient derived SCZ models. Regulation of microtubule polymerization is amongst the top categories when differentially regulated genes of SCZ endothelial cells are categorized based on biological function. Previous studies have shown that proper functioning of the endothelial barrier in various tissues is associated with correct cytoskeletal reorganization, the activation of actomyosin contraction and gap junction formation [96, 119]. In quiescent endothelial cells, microtubule density is the highest in the centrosome region and dissipates as one moves to cell margins, making microtubule organization highly spatially and temporally controlled [119]. It would be interesting to study the regulation of microtubule polymerization in induced BMECs of SCZ and see if modulating microtubule density can lead to changes in endothelial properties such as gap or tube formation. Consequently, this could alter properties such as permeability or barrier formation which our study demonstrates as possible SCZ endothelial cell phenotypes.

## Materials and methods

### Human iPSC lines

Human iPSC lines were obtained from NIH repositories at the Rutgers University Cell and DNA Repository. All lines have undergone extensive characterization for identity, pluripotency, exogenous reprogramming factor expression, genetic stability, and viability. A total of 23 different iPSC lines (8 CTRL, 15 SCZ) were utilized. All of the lines used in this study were previously published [13]. A comprehensive list and description of SCZ patient iPSC donors and their clinical characteristics are provided in Supplementary Table 1 of Notaras et al., 2022 and Supplementary Table 2 in this study. All SCZ samples were derived from idiopathic cases that maintained unknown disease origins and do not meet a genetic/syndrome-based diagnosis (as listed in NIMH notes) that would otherwise explain ontogeny of disease. CTRL iPSC lines were screened for both personal, and family history, of major mental illnesses (Supplementary Table 1). No sex difference in phenotype was observed between iPSC lines (See Supplementary Table 3 for the list of the lines used in each experiment). All but one of the donors were adults at the time of biopsy (range: 9, 29, 30,36, 46, and 58 years of age), alas no age-mediated differences between the iPSC lines were identified in any experimental assay or in quality control assessments. All iPSC lines were maintained on Vitronectin-coated plates and fed with Essential 8 (E8) + E8 supplement (ThermoFisher, CAT#: A1517001). Human iPSC lines were cultured simultaneously to control for idiosyncratic culturing conditions. iPSC lines had typically undergone extensive standardized testing for common iPSC factors such as: pluripotency, viability, and karyotypes. In all experiments, low passages (less than 18) were used, and all differentiations were derived from a single clone for each line. In total, 25 independent iPSC lines were sampled across experiments and have undergone extensive standardized testing described above before being included in the manuscript.

### Three-dimensional cerebral organoid culturing system

Recent organoid models often apply exogenous growth factors (e.g., BDNF, NT-3, EGF and/or pathway modulators (e.g., TGFβ and WNT pathway inhibitors) to direct the differentiation of progenitors towards a neuronal fate or promote their survival by reducing apoptosis. These extrinsic patterning and survival factors yield more uniform organoids, and they are especially useful for unraveling organoid biology and the mechanisms of corticogenesis in healthy systems. However, factors such as BDNF and related neurotrophins, TGFβ, and WNT signaling are longstanding contributors to SCZ pathophysiology and may play a role in the ontogenesis of disease during early neurodevelopment. Indeed, these same factors and pathways support hypotheses on early disease development via their effects on stem cells and neuronal programming. In fact, scRNA-seq analysis revealed that progenitors in SCZ organoids (generated via morphogen-free protocol) exhibited down-regulated *BDNF* and *TrkB*, but up-regulated *NT-4*, gene-expression [13], pointing to the importance of organoid protocol selection to fully detect disease phenotypes. Therefore, we adapted a morphogen-free organoid protocol for our primary experiments to avoid these potential confounding factors. We adapted the morphohen-free organoid protocol that was previously published [37, 38]. This culturing system can be minimized into four major stages. First, iPSCs are dissociated with Accutase (Laboratory Disposable Products, CAT#: 25-058-C1) and cultured into three-dimensional embryoid bodies for up to 7 days using previously described media [38] in ultra-low attachment 96 well plates (Corning; CAT#: 3474). Rock inhibitor (1:1000; Stem Cell Tech, CAT#: 72304) and basic fibroblast growth factor (Pepro Tech, CAT#: 100-18B) are included in media for the first 2–4 days of embryoid body culturing to promote stem cell aggregation and survival. Following this, healthy embryoid bodies are isolated and transferred to Nunclon Sphera 24 well plates (Thermo Scientifiic, CAT#: 174930) for neural fate specification, using custom neural induction media [38]. Once neuroepithelium was apparent, successful early “organoids” were embedded in a 30 µL Matrigel (Corning, CAT#: 354234) spheroid-droplet and polymerized at 37 °C for 20–30 min which provided a matrix for subsequent neural expansion. Organoids suspended in matrigel droplets were next cultured in terminal organoid media [38] for 4–6 days without agitation, and then cultured with agitation at 60–70 RPM until harvested for experiments. All stages of culturing occurred at 37 °C with 5% atmospheric CO2 in a sterile incubator. Cerebral organoids from healthy CTRL and SCZ iPSC lines were generated and maintained in parallel, ensuring that idiosyncratic differences in culturing conditions were accounted for between CTRL and SCZ organoids. Last, all cultures underwent quality control assessments on a rolling basis, utilizing the published criterion and guidelines [38] which describes numerous “Go/No Go” criteria at different stages of organoid generation. Independent confirmation of neuronal induction was also achieved via unbiased analysis of our scRNA-Seq and proteomics datasets [13]. In addition to establishing organoid reproducibility via computational analysis [13], we have also verified organoid reproducibility with extensive measurements of proliferation, progenitor pools, neuronal induction, and cell death to confirm low baseline variability (Supplementary Fig. 1).

We have utilized the same go-no-go quality control criteria published for our relevant organoid generation protocol in Nature [37] and Nature Methods [38]. First, only organoids that exhibited morphological evidence of neural induction were advanced in our organoid-culturing pipeline as outlined in the quality control steps from adapted protocols. This included assessment for the presence of homotypic morphological markers that are indicative of neuroepithelial expansions, neuroectoderm induction, and ventricular zone formation via light microscopy. This allowed for confirmation of neural stem cell expression (NESTIN+, SOX2+ cells), forebrain-specific progenitor induction (FOXG1+, PAX6+ cells), and evidence of neuronal induction (MAP2+, DCX+ cells) including forebrain-specific neuronal induction (CTIP2+, BRN2+ cells). As an unbiased confirmation, our multi-omics data ensured that our adaptation of published go-no-go criteria yielded sufficient neural induction within organoids, as 17 of 22 of our most up-regulated gene ontology pathways mapped to neural development [13].

### Single-cell RNA sequencing

To define cell-specific transcriptomes in CTRL and SCZ organoids, we performed scRNA-seq. First, organoids from 3 CTRL and 3 SCZ lines were dissociated using Accutase, filtered to remove debris and subjected to high-throughput FACS (Aria II flow cytometer, Becton Dickson). This allowed for isolation of only live cells at concentration of 2000 live cells/µL, standardizing microfluidic-device loading between lines. Cell viability was confirmed using Countess-II (Invitrogen, Thermofisher, CAT#: AMQAX1000). Next, live cell suspensions we rapidly loaded into 10x chromium microfluidic devices to produce barcoded single-cell nanodroplet emulsions and cells were prepared for 10X Genomics Chromium library preparation according to manufacturer instructions (ChromiumTM Single-Cell 3’ Library and Gel Beads and ChromiumTM CHIP kit, 10x Genomics). After sample generation, barcoded-emulsions were broken, amplified, and libraries prepared. cDNA was examined on a Fragment Analyzer running PROSize v3.0 3.0.1.6 (Advanced Analytical Technologies). Libraries were subsequently subjected to next generation sequencing via Next-Seq High-Output 150-cycled 26-8-9. Raw reads were processed after sequencing as subsequently described.

### Bioinformatics pipeline for single-cell RNA sequencing

After sequencing, raw reads were aligned to GRCh38 with the CellRanger v3.0.2 pipeline (10x Genomics, USA). Subsequent analyses of quality control and filtering, data integration and normalization, clustering and visualization were conducted using Seurat (v. 3.1). Further quantification and statistical analyses were performed in R. To remove outliers, cells with less than 200 genes and >10% mitochondrial reads were filtered out. Read counts were individually normalized using SCT (SCTransform) for each sample. The different samples were then integrated into a Seurat object using the top 3000 most informative genes prior to performing various dimensionality reduction steps, which include principal components analysis (PCA) and uniform manifold approximation and projection (UMAP). A shared nearest neighbor graph was constructed with default settings (e.g., *k* = 20), which comprised the first 30 principal components. Subsequent clustering was performed with the resolution parameter set to 0.1. Gene set enrichment and overrepresentation analyses were performed using WebGestalt and EnrichR. Genes or terms were ranked based on the adjusted *p*-value (Benjamini–Hochberg) and significantly affected gene sets were selected based on an adjusted *p*-value of < 0.05. In the gene set analysis for endothelial clusters, Ingenuity Pathway Analysis (IPA) and GeneAnalytics were used to investigate the differentially expressed genes. We utilized Ingenuity Pathway Analysis (IPA) software to identify the top canonical pathways associated with our data set as well as potential upstream regulators. We focused on the significant pathways leveraging the ‘KEGG 2016’ functional database. We also included ‘Jensen tissues’ for statistically associating our gene sets to corresponding human tissues and ‘Descartes 2021’ for single-cell gene expression profiling of cell types and tissues. Moreover, we included significant results for ‘TF-Gene’ co-occurrence analysis investigating genes most likely to co-occur with transcription factors based on a large-scale gene co-occurrence adjacency matrix. Brain-specific functional module prediction was conducted utilizing a shared k-nearest neighbor approach to obtain a tissue specific network. The Louvain algorithm further clustered this network into specific modules and the associated Q values for each term were calculated using one-sided Fisher’s exact tests and subsequent Benjamini–Hochberg corrections to correct for multiple testing.

### Immunohistochemistry and laser-scanning confocal microscopy

In preparation for immunohistochemistry, organoids were drop-fixed in 4% paraformaldehyde, dehydrated in 30% sucrose and embedded in Tissue-Tek using OCT compound (CAT#: 4583) and biopsy molds. Organoids were serially cryosectioned between slides at 30 µm. Each slide from this sectioning thus contained 3-4 unique sections/Fields of View (FOV) per each organoid studied. Using this approach, we were able to robustly assess both independent and focal cell populations in each biological and technical organoid replicate. In further preparation all sections underwent heat-mediated antigen retrieval in the citrate buffer and primary antibody was incubated for each section overnight. Primary antibodies comprised PECAM1 (1:200, ThermoFisher, CAT#: # MA3100) MAP2 (1:1000, Abcam, CAT#: AB11267), pH3 (1:1000; Millipore, CAT#: 06-570), Ki67 (1:1000; BD Biosciences, CAT#: 550609). Secondary antibodies were incubated for 2 h at room temperature and comprised antibodies for rabbit (Fluor 488 CAT#: A11008; Fluor 546 CAT#: A11035; & Fluor 633 CAT#: A21070), mouse (Fluor 488 CAT#: A11001; Fluor 546 CAT#: A11003; & Fluor 633 CAT#: A21052) and chicken (Fluor 546 CAT#: A11040) were used at a 1:2000 dilution and sourced from Life Technologies. Microscopy was completed on an Olympus IX81 Laser-Scanning Confocal Microscope, controlled by proprietary Olympus FluoView software. Images were typically acquired at 1200 × 1200 resolution with optical *Z* slices (step sizes) ranging from 0.5 to 10 µm depending on the unit of analysis. In total, 8 CTRL and 15 SCZ patient lines were used for analysis. For counting microvascular-like vessels, images were converted to 8-bit and skeletonized using ImageJ. A skeleton-branch was defined as a microvascular-like vessel if it was longer than 1 µm and for each line vessels were counted from at least 5 different organoids per each line.

### Differentiation of iPSCs to brain microvascular endothelial cells

3 independent CTRL (MH0159021, MH0159020, MH0159679) and 3 independent SCZ (MH0185964, MH0200865, MH0159025) iPSCs lines were differentiated into endothelial cells as described previously [79]. All cell lines were maintained between passages 10 to 40 on Matrigel (BD Biosciences, 354277) in mTeSR (STEMCELL Technologies, 85850) with 10 µM Y-27632 (ROCK inhibitor). To start differentiation, cells were passaged onto Matrigel in mTeSR1 medium and allowed to expand until confluent (usually 3 days: Day -3). Approximately 24 h later (i.e., Day -2), mTeSR1 + ROCK inhibitor was replaced with 2 mL mTeSR1 in each well and same was done in Day -1 as well. Cultures were then switched to unconditioned medium (Day 0) for 5 days (unconditioned medium: DMEM/F12 Basal Media, 20% Knock-out Serum Replacement, 1× MEM nonessential amino acids, 0.5× Glutamax, 0.1 mM of 55 mM beta-mercaptoethanol). Medium was changed every day during the 5 days. On Day 6, medium was switched to Serum Free Endothelial Cell Media and transduced with lentiviral ETV2, ERG and FLI1 and cultured for 2 days (Serum Free Endothelial Cell Media: EC serum free medium Gibco cat# 11111044, 20 ng/mL bFGF Peprotech cat# 100-18B, 1% platelet-poor plasma-derived bovine serum Alfa Aesar cat# J64483AE, and 10 μM retinoic acid Sigma cat# R2625). On Day 8, cells were dissociated with Accutase (Invitrogen, 004555-56) and plated onto six-well polystyrene plates coated with a mixture of Collagen IV (400 mg/mL; Sigma, C6745) and Fibronectin (100 mg/mL; Sigma, F1141) in Serum Free Endothelial Cell Media. Cells were fed every 2 days until they were 80% confluent; then prepared for FACS isolation. Cells were FACS isolated based on PECAM1+, CDH5+, EPCAM- staining and cultured on 0.1% gelatin plates in endothelial media (+2% FBS) for expansion.

### Dextran cell permeability assay

CTRL and SCZ iBMECs were passaged and cultured as a monolayer (100,000 cells/well) onto Transwell plates with 0.4-μm pore sized inserts (Corning). Cells were grown until confluency at which time 50 ng/mL anti-VE-cadherin antibody (BV9) was added to the cell culture media. Seventy kilodaltons of FITC-labeled dextran (Invitrogen) was then pipetted into each well and allowed to incubate for 6 h. The amount of dextran that passed through the cell monolayer was measured by collecting the media passed through the Transwell filter and analyzing it on a spectrophotometer (Bio-Rad). Dextran intensity graphs were created using Prism.

### Statistical analysis

All statistical tests and graphing were performed within the R statistical environment (v.4.2.2) and Graphpad Prism (v.10.1.0). All data is presented as Mean ± Standard Error of the Mean (SEM). To determine differentially expressed genes (DEGs), non-parametric Wilcoxon rank sum tests were performed as part of the Seurat (v. 3.1) package. For gene set enrichment analyses, WebGestalt software was utilized on the DEGs and determined a high-level summary of biological categories based on the Gene Ontology (GO) terms including identifying relevant modules pertaining to Biological Process, Molecular Function and Cellular Component. Ingenuity Pathway Analysis (IPA) of top canonical biological signaling pathways using the DEGs between schizophrenia cases and controls as the input was calculated based on an underlying hypergeometric distribution, where the negative logarithm of the significance level is obtained by Fisher’s exact test at the right tail. For transcription factor (TF) co-occurrence analysis we leveraged the co-occurrence adjacency matrix from TF-gene pairings from EnrichR. Brain-specific functional module prediction was conducted utilizing a shared k-nearest neighbor approach to obtain a tissue specific network. The Louvain algorithm further clustered this network into specific modules and the associated Q values for each term were calculated using one-sided Fisher’s exact tests. Benjamini–Hochberg corrections were performed to correct for multiple testing in these predicted functional modules. Upstream regulator analysis was also conducted in IPA in order to identify the molecules upstream of the implicated genes from the endothelial population enrichment analyses. This entailed testing the overlap of DEGs with network-related genes against a random model and determining which network connections are unlikely to occur, yielding a p-value and an activation Z-score. These results were then used to prioritize the likely regulating molecules. As most comparisons downstream of RNA sequencing experiments comprised only two groups, the Student’s *t*-test was the predominant hypothesis-test utilized for both immunohistochemistry analysis and dextran permeability assay. Significance was set at *p* < 0.05 per Fisher’s tables, tailed according to statistical-directionality guidelines and corrected for multiple-comparisons. For immunohistochemistry imaging, a minimum of 5 organoids and up to 15 fields of vision were quantified for each line presented (*n* = 23 lines). In the dextran permeability assay, 3 biological replicates (lines)/per group and 3 technical replicates/per line were analyzed.

### Supplementary information

Supplementary Material

## Data Availability

The scRNA-seq data will be submitted to the NIH dbGaP.
