# Supplementary material for: Schizophrenia endothelial cells exhibit higher permeability and altered angiogenesis patterns in patient-derived organoids
Source: Transl Psychiatry. 2024 Jan 23;14:53. doi: 10.1038/s41398-024-02740-2 (PMC10806043; doi:10.1038/s41398-024-02740-2)
Supplement: Supplementary file 1 — Supplementary Material [file 41398_2024_2740_MOESM1_ESM.docx]

**SUPPLEMENTARY MATERIAL FOR:**

**Schizophrenia endothelial cells exhibit higher permeability and altered angiogenesis patterns in patient-derived organoids**

Isidora Stankovic^1^, Michael Notaras^1^, Paul Wolujewicz^1^, Tyler Lu^2,3^, Raphael Lis^2,3^, M. Elizabeth Ross^1^, Dilek Colak^1,4,*^

1. Center for Neurogenetics, Feil Family Brain and Mind Research Institute, Weill Cornell Medicine, Cornell University, New York, NY, USA

2. Ansary Stem Cell Institute, Division of Regenerative Medicine, Department of Medicine, Weill Cornell Medicine, New York, NY, USA

3. Ronald O. Perelman and Claudia Cohen Center for Reproductive Medicine, Weill Cornell Medicine, New York, NY, USA

4. Gale and Ira Drukier Institute for Children’s Health, Weill Cornell Medicine, Cornell University, New York, NY, USA

*Correspondence to Dilek Colak at **dic2009@med.cornell.edu**

**Supplementary Information**

Supplementary information includes 4 tables and 2 Figures.

**Supplementary Tables**

**Supplementary Table 1:** Clinical notes of NIMH and Coriell CTRL iPSC lines

**Supplementary Table 2:** Clinical notes of NIMH SCZ iPSC lines

**Supplementary Table 3:** CTRL and SCZ iPSC lines used in each experiment

**Supplementary Table 4:** 260 genes that are significantly differentially regulated between EC cluster of SCZ and CTRL organoids

**Supplementary Figures**

**Supplementary Figure 1:** Variability of cerebral organoids generated by morphogen-free protocol

**Supplementary Figure 2:** Data split analyses by individual CTRL and SCZ lines for quantifications in Figure 1 and Figure 5

**Supplementary Table 1. Clinical notes of NIMH and Coriell CTRL iPSC lines:** All controls had no history or reported family history of SCZ*.

| **CTRL iPSC Line** | **Sex** | **Ethnicity** | **Age of Sampling** |
| --- | --- | --- | --- |
| MH0159019 | Female | Hispanic | 29 |
| MH0159020 | Male | White | 58 |
| MH0159021 | Male | Hispanic | 32 |
| MH0174677 | Male | White | 9 |
| MH0174679 | Male | White | 17 |
| MH0174686 | Male | White | 17 |
| GM23279 | Female | White | 36 |
| GM25256 | Male | Asian | 30 |

All human iPSCs were purchased from deposits made to the National Institute of Mental Health (NIMH) Repository & Genomics Resource Center at Rutgers University (lines beginning with prefix MH) or from the Coriell Institute for Medical Research (lines beginning with prefix GM).

**Supplementary Table 2. Clinical notes of NIMH SCZ iPSC lines***

| **SCZ iPSC Line** | **Sex** | **Ethnicity** | **Age of Sampling** | **Age of Onset** | **Clinical Notes** |
| --- | --- | --- | --- | --- | --- |
| MH0159025 | Male | White | 48 | 41 | Paranoid SCZ. Cannabis & alcohol abuse, drug overdose history, father had depression/drug abuse history, one sibling has bipolar disorder, behavioral problems from age 10. |
| MH0159026 | Male | White | 60 | 38 | Persistent auditory hallucinations, lifetime cannabis & alcohol abuse, nicotine addiction, brother had schizophrenia. |
| MH0185923 | Male | White | 26 | - | Episodes of agitation, delusions of persecution, and fear of assassination; at age four mild features of pervasive developmental disorder, SCZ/SA/ASD father and sister, brother autistic at age four |
| MH0185225 | Male | White | 23 | - | Paralogical thinking, affective shielding, splitting of affect from content, suspiciousness, SCZ/SPD father, anorexic/schizoid sister. |
| MH0200865 | Male | White | 25 | 12 | Childhood onset. Persistent delusional thoughts, persecutory auditory hallucinations, impulsive and hyperactive behavior. Autistic brother. Psychosis scores at baseline (off meds): SAPS = 29, SANS = 49, BPRS24 = 87. |
| MH0217268 | Female | White | 49 | - | - |
| MH0185900 | Male | White | 31 | - | PANSS Total = 71 |
| MH0185954 | Female | Mixed (Non Hispanic) | 34 | - | PANSS Total = 100 |
| MH0185963 | Female | Black | 47 | - | PANSS Total = 71 |
| MH0185970 | Male | White | 52 | - | PANSS Total = 105 |
| MH0185912 | Male | White | 34 | - | PANSS Total = 106 |
| MH0185945 | Male | Hispanic | 58 | - | PANSS Total = 78 |
| MH0185964 | Female | Asian | 43 | - | PANSS Total = 72 |
| MH0185925 | Male | Mixed (Null) | 64 | - | PANSS Total = 62 |
| MH0185928 | Female | Mixed (Non Hispanic) | 47 | - | PANSS Total = 88 |

* Clinical psychometrics and age of onset provided only if known. No further information regarding mixed races was provided from the NIMH beyond the disclaimer that these individuals comprise more than one race. Mixed races were further categorized as “Hispanic” vs. “Non-Hispanic” or “Null” (missing or unknown). The Positive and Negative Syndrome Scale (PANSS) is used in clinical trials of SCZ and is considered the “gold standard” for assessment.

**Supplementary Table 3: CTRL and SCZ lines used in specific experiments***

| **Experiment** | **Sampling** | **Specific NIMH iPSC Donors** |
| --- | --- | --- |
| Single-cell RNA sequencing | n = 6 iPSCs (3 CTRL, 3 SCZ)  n = 26,335 transcriptomes and n = 20,844 genes profiled in sum | **CTRL:** MH0159019, MH0159020, MH0159021,  **SCZ:** MH0159025, MH0159026, MH0200865 |
| Immunohistochemical visualization of ECs | N = 23 iPSCs (8 CTRL, 15 SCZ) | **CTRL:** All in Table S1 **SCZ:** All in Table S2 |
| Differentiation of iBMEC and dextran permeability assay | N = 6 iPSCs (3 CTRL, 3 SCZ) | **CTRL:** MH0159020, MH0159021, MH0174679  **SCZ:** MH0185964, MH0200865, MH0159025 |

* At the time we performed the scRNA-seq experiments for our previous study using the SCZ lines [13], we were restricted by the number of the patient lines that were available. Thus, initially, both for proteomics and scRNA-seq experiments, we used 3 lines per group. During the manuscript/publication phase of Notaras *et al*., *Molecular Psychiatry* 2022, we obtained additional SCZ lines. To confirm the endothelial cell number phenotype in SCZ organoids, we tried to use as many lines as possible during PECAM immunohistochemistry experiments. 15 out of 17 SCZ lines generated high quality organoids. Therefore, we were able to perform immunohistochemistry in organoids derived from 15 SCZ lines (which also included all 3 SCZ lines used for scRNA-seq) for endothelial cell quantification. While we also intended to perform the dextran assay with as many lines as possible, induced BMEC differentiation is challenging, and some lines are resistant to yielding uniform BMEC populations. Although we started with 6 lines per group, only 3 lines per group yielded high quality BMEC populations as characterized by FACS. 2 of the 3 lines per group used for BMEC differentiation, were the same lines used for scRNA-seq experiments.

**Supplementary Table 4: 260 genes that are significantly differentially regulated between EC cluster of SCZ and CTRL organoids**

| **Gene** | **p_val** | **avg_log2FC** | **pct.1** | **pct.2** | **p_val_adj** |
| --- | --- | --- | --- | --- | --- |
| CHCHD2 | 5.77E-31 | -5.7367305 | 0.232 | 0.841 | 1.35E-26 |
| TMSB4X | 2.40E-29 | -305.86344 | 0.995 | 1 | 5.62E-25 |
| MEG3 | 6.42E-25 | -23.939176 | 0 | 0.171 | 1.50E-20 |
| SOX11 | 1.31E-24 | -11.490751 | 0.133 | 0.585 | 3.07E-20 |
| MARCKSL1 | 6.22E-22 | -37.528271 | 0.985 | 1 | 1.46E-17 |
| NREP | 2.33E-21 | -5.28354 | 0.553 | 0.927 | 5.46E-17 |
| DCX | 2.73E-21 | -1.5041299 | 0.002 | 0.159 | 6.39E-17 |
| HBZ | 8.30E-20 | Inf | 0.691 | 0.171 | 1.94E-15 |
| IL32 | 6.14E-19 | -9.4110978 | 0.08 | 0.402 | 1.44E-14 |
| TMSB15A | 1.24E-18 | -14.936607 | 0.087 | 0.415 | 2.89E-14 |
| MLLT11 | 5.11E-18 | 7.18483836 | 0.27 | 0.683 | 1.20E-13 |
| CDKN1A | 6.34E-18 | 6.76318002 | 0.164 | 0.549 | 1.48E-13 |
| FAM213A | 6.94E-18 | -6.4442079 | 0.471 | 0.878 | 1.62E-13 |
| PLK2 | 8.38E-18 | -7.035043 | 0.404 | 0.756 | 1.96E-13 |
| PGF | 3.20E-17 | -9.4951883 | 0.023 | 0.232 | 7.50E-13 |
| GNG2 | 3.73E-17 | -6.3888217 | 0.113 | 0.451 | 8.74E-13 |
| TCEAL7 | 1.47E-16 | -2.982179 | 0.005 | 0.146 | 3.45E-12 |
| ZIC1 | 1.79E-16 | -0.4430484 | 0 | 0.11 | 4.20E-12 |
| TTR | 2.00E-16 | -0.7347637 | 0.031 | 0.256 | 4.67E-12 |
| STMN4 | 2.77E-16 | -1.7895804 | 0.002 | 0.122 | 6.48E-12 |
| LAMA4 | 3.91E-16 | -0.6219774 | 0.025 | 0.232 | 9.15E-12 |
| CRHBP | 5.24E-16 | 54.3571292 | 0.614 | 0.085 | 1.23E-11 |
| TMEM178A | 8.18E-16 | -1.063643 | 0.008 | 0.159 | 1.92E-11 |
| EIF4G2 | 1.12E-15 | -9.9990399 | 0.714 | 0.951 | 2.62E-11 |
| KIF5C | 2.23E-15 | -3.9097978 | 0.011 | 0.171 | 5.23E-11 |
| PCSK1N | 7.89E-15 | -2.9577452 | 0.01 | 0.159 | 1.85E-10 |
| PAX6 | 1.37E-14 | -0.8193522 | 0.002 | 0.11 | 3.22E-10 |
| RPS21 | 1.79E-14 | 60.5923931 | 0.984 | 0.927 | 4.20E-10 |
| MALAT1 | 1.84E-14 | 155.798978 | 0.993 | 0.988 | 4.30E-10 |
| MT-ATP6 | 5.05E-14 | 1.40442352 | 0.915 | 0.976 | 1.18E-09 |
| YBX1 | 5.69E-14 | 20.1864395 | 0.971 | 1 | 1.33E-09 |
| GTF2H2 | 1.15E-13 | -0.3838593 | 0.028 | 0.22 | 2.69E-09 |
| ARHGAP18 | 1.70E-13 | -1.9542959 | 0.029 | 0.22 | 3.99E-09 |
| TTYH1 | 1.97E-13 | -2.696265 | 0.005 | 0.122 | 4.62E-09 |
| MT-ND2 | 3.79E-13 | -38.537784 | 0.889 | 0.976 | 8.87E-09 |
| BST2 | 3.79E-13 | 10.0883485 | 0.81 | 0.39 | 8.88E-09 |
| EMX2 | 4.14E-13 | -0.6952131 | 0.003 | 0.11 | 9.70E-09 |
| F2RL3 | 4.22E-13 | -0.4941723 | 0.003 | 0.11 | 9.89E-09 |
| MT-ND3 | 4.54E-13 | -10.181877 | 0.864 | 0.951 | 1.06E-08 |
| C1orf61 | 6.49E-13 | -15.174157 | 0.056 | 0.28 | 1.52E-08 |
| MT-ND5 | 9.41E-13 | -10.27658 | 0.561 | 0.817 | 2.20E-08 |
| RPS27L | 1.27E-12 | -3.96759 | 0.779 | 0.915 | 2.97E-08 |
| TUBB2B | 1.54E-12 | -11.013812 | 0.214 | 0.537 | 3.61E-08 |
| BTG1 | 1.80E-12 | -3.7258525 | 0.684 | 0.927 | 4.21E-08 |
| TTC3 | 1.90E-12 | -7.9061615 | 0.442 | 0.78 | 4.44E-08 |
| HOXD1 | 2.40E-12 | -0.8972154 | 0.015 | 0.159 | 5.62E-08 |
| SULF2 | 2.66E-12 | -1.0396474 | 0.038 | 0.232 | 6.23E-08 |
| CNTNAP2 | 2.71E-12 | -0.8350772 | 0.007 | 0.122 | 6.34E-08 |
| ID4 | 2.93E-12 | -0.4470813 | 0.018 | 0.171 | 6.87E-08 |
| APLN | 2.93E-12 | -1.754521 | 0.018 | 0.171 | 6.87E-08 |
| IFITM3 | 3.94E-12 | 51.0280028 | 0.98 | 0.89 | 9.21E-08 |
| CAVIN2 | 3.98E-12 | -5.1444113 | 0.157 | 0.451 | 9.31E-08 |
| OCIAD2 | 4.31E-12 | -2.0312814 | 0.118 | 0.39 | 1.01E-07 |
| CD93 | 5.46E-12 | -29.558562 | 0.278 | 0.573 | 1.28E-07 |
| NETO2 | 1.27E-11 | -3.0775036 | 0.173 | 0.488 | 2.98E-07 |
| CDC42EP3 | 1.95E-11 | -4.3120552 | 0.124 | 0.39 | 4.56E-07 |
| TMEM255B | 2.47E-11 | -1.6341433 | 0.162 | 0.451 | 5.79E-07 |
| SRGAP3 | 2.87E-11 | -0.5437973 | 0.008 | 0.122 | 6.73E-07 |
| TUBA1A | 3.42E-11 | -23.098653 | 0.679 | 0.878 | 8.01E-07 |
| AFP | 5.37E-11 | -0.3364052 | 0.011 | 0.134 | 1.26E-06 |
| MAP1B | 5.58E-11 | -31.745659 | 0.47 | 0.732 | 1.31E-06 |
| SLC38A1 | 5.60E-11 | -0.9593958 | 0.059 | 0.268 | 1.31E-06 |
| TRIB2 | 6.57E-11 | -0.5143732 | 0.029 | 0.195 | 1.54E-06 |
| RPL7 | 6.60E-11 | 44.7112815 | 0.998 | 1 | 1.54E-06 |
| EPS8L1 | 7.32E-11 | -0.5201591 | 0.018 | 0.159 | 1.71E-06 |
| ARHGAP29 | 7.88E-11 | -7.0885115 | 0.448 | 0.695 | 1.85E-06 |
| STMN1 | 9.26E-11 | 23.0709686 | 0.956 | 1 | 2.17E-06 |
| SFRP1 | 1.06E-10 | -13.971462 | 0.072 | 0.293 | 2.48E-06 |
| MIAT | 2.08E-10 | -9.4830333 | 0.013 | 0.134 | 4.87E-06 |
| SRP9 | 2.11E-10 | -1.5386533 | 0.727 | 0.963 | 4.95E-06 |
| ZFHX4 | 2.67E-10 | -0.4092875 | 0.016 | 0.146 | 6.24E-06 |
| FGF13 | 2.84E-10 | -0.3478098 | 0.016 | 0.146 | 6.64E-06 |
| IFT57 | 3.80E-10 | -1.3329555 | 0.103 | 0.341 | 8.91E-06 |
| RPS29 | 4.50E-10 | 102.419261 | 1 | 1 | 1.05E-05 |
| FHL1 | 5.75E-10 | -1.2428295 | 0.049 | 0.232 | 1.35E-05 |
| IGFBP2 | 6.55E-10 | -10.557398 | 0.946 | 0.988 | 1.53E-05 |
| CRABP1 | 6.56E-10 | -33.165278 | 0.1 | 0.329 | 1.54E-05 |
| ELP5 | 7.14E-10 | -0.5462983 | 0.113 | 0.366 | 1.67E-05 |
| HLX | 7.21E-10 | -1.1076629 | 0.16 | 0.439 | 1.69E-05 |
| CHST1 | 7.35E-10 | -21.643308 | 0.116 | 0.354 | 1.72E-05 |
| KCNJ2 | 7.40E-10 | -0.5855516 | 0.021 | 0.159 | 1.73E-05 |
| FJX1 | 7.86E-10 | -6.3331577 | 0.157 | 0.427 | 1.84E-05 |
| SYT1 | 8.00E-10 | -0.889232 | 0.008 | 0.11 | 1.87E-05 |
| STMN2 | 8.72E-10 | 23.0358972 | 0.029 | 0.183 | 2.04E-05 |
| SH3BP5 | 1.06E-09 | -1.8982879 | 0.111 | 0.354 | 2.47E-05 |
| ABCA9-AS1 | 1.15E-09 | -0.9473282 | 0.015 | 0.134 | 2.70E-05 |
| FAM107B | 1.46E-09 | -6.0195669 | 0.37 | 0.646 | 3.42E-05 |
| TP53I11 | 1.46E-09 | -8.6885441 | 0.339 | 0.646 | 3.42E-05 |
| H2AFZ | 1.49E-09 | -44.540358 | 0.882 | 1 | 3.49E-05 |
| RNF144A | 1.50E-09 | -0.766368 | 0.111 | 0.354 | 3.51E-05 |
| DCK | 1.93E-09 | -0.4709494 | 0.083 | 0.305 | 4.51E-05 |
| H3F3B | 2.49E-09 | -7.1455406 | 0.997 | 0.988 | 5.82E-05 |
| ITGA2 | 2.60E-09 | -0.3444606 | 0.023 | 0.159 | 6.09E-05 |
| FKBP1A | 2.97E-09 | -14.44745 | 0.949 | 0.878 | 6.96E-05 |
| RALGAPA1 | 3.03E-09 | -0.3906642 | 0.041 | 0.207 | 7.10E-05 |
| IGFBP5 | 3.46E-09 | -11.826382 | 0.059 | 0.244 | 8.10E-05 |
| OSTF1 | 3.62E-09 | -0.8719702 | 0.111 | 0.341 | 8.48E-05 |
| TNFAIP8L1 | 3.66E-09 | -1.7066949 | 0.119 | 0.354 | 8.58E-05 |
| PPP4C | 3.68E-09 | -1.7084112 | 0.422 | 0.683 | 8.62E-05 |
| DAPL1 | 3.69E-09 | -0.5012949 | 0.02 | 0.146 | 8.63E-05 |
| HYAL2 | 3.96E-09 | -4.8829116 | 0.435 | 0.671 | 9.27E-05 |
| BAZ1B | 4.24E-09 | -0.7625419 | 0.116 | 0.354 | 9.93E-05 |
| ZFP36L2 | 4.30E-09 | -2.094012 | 0.183 | 0.439 | 0.00010071 |
| ADGRF5 | 5.12E-09 | -1.1744941 | 0.106 | 0.329 | 0.00011992 |
| AEN | 5.20E-09 | -0.6406408 | 0.064 | 0.256 | 0.00012168 |
| TIGAR | 5.28E-09 | -0.4258491 | 0.047 | 0.22 | 0.00012369 |
| RPL28 | 5.33E-09 | 107.216222 | 1 | 1 | 0.00012488 |
| LEF1 | 5.47E-09 | -0.745428 | 0.083 | 0.293 | 0.00012802 |
| CXCR4 | 6.18E-09 | -5.2689525 | 0.095 | 0.305 | 0.00014469 |
| GABARAPL2 | 6.96E-09 | -4.9122222 | 0.547 | 0.768 | 0.00016301 |
| APCDD1 | 8.35E-09 | -0.5136132 | 0.049 | 0.22 | 0.00019543 |
| ZNF146 | 8.55E-09 | -1.4883639 | 0.142 | 0.39 | 0.00020027 |
| CASC15 | 8.86E-09 | -0.9468456 | 0.029 | 0.171 | 0.00020751 |
| ZFP36L1 | 8.93E-09 | -4.9314431 | 0.663 | 0.854 | 0.00020904 |
| MT-ND4 | 8.97E-09 | -28.936084 | 0.98 | 0.988 | 0.00020996 |
| RGCC | 9.25E-09 | -1.2807221 | 0.085 | 0.293 | 0.00021657 |
| ERG | 9.27E-09 | -1.3705311 | 0.175 | 0.439 | 0.00021697 |
| LINC00632 | 9.53E-09 | -0.4580743 | 0.029 | 0.171 | 0.00022311 |
| CUL3 | 1.11E-08 | -0.7853562 | 0.121 | 0.354 | 0.00026034 |
| CDK2AP1 | 1.62E-08 | -3.0020066 | 0.231 | 0.488 | 0.00037994 |
| ADGRL4 | 1.76E-08 | -4.5173422 | 0.254 | 0.5 | 0.00041198 |
| COL4A1 | 1.85E-08 | -8.8940796 | 0.363 | 0.622 | 0.00043334 |
| NUDT14 | 1.92E-08 | -1.7834938 | 0.149 | 0.39 | 0.00044942 |
| HNRNPU | 1.99E-08 | 0.3618289 | 0.542 | 0.817 | 0.00046601 |
| HES4 | 2.08E-08 | -3.202568 | 0.229 | 0.5 | 0.00048658 |
| PCDH1 | 2.11E-08 | -1.0094085 | 0.052 | 0.22 | 0.00049375 |
| KCTD3 | 2.17E-08 | -0.4256754 | 0.051 | 0.22 | 0.00050881 |
| BCL6B | 2.24E-08 | -8.8221344 | 0.278 | 0.549 | 0.00052372 |
| MORF4L2 | 2.41E-08 | -0.5584965 | 0.64 | 0.866 | 0.00056485 |
| RGS16 | 2.80E-08 | -1.4756041 | 0.047 | 0.207 | 0.00065483 |
| EIF2AK1 | 2.90E-08 | -0.6756637 | 0.111 | 0.329 | 0.00067866 |
| TMEM230 | 3.08E-08 | -5.1987906 | 0.566 | 0.756 | 0.00072063 |
| TMEM184B | 3.14E-08 | -0.9628789 | 0.082 | 0.28 | 0.00073482 |
| RAPGEF5 | 3.28E-08 | -1.453613 | 0.093 | 0.293 | 0.00076727 |
| MT-CYB | 3.48E-08 | -63.490515 | 0.967 | 0.988 | 0.0008153 |
| RAB6A | 3.94E-08 | -2.3614868 | 0.37 | 0.646 | 0.00092164 |
| YWHAH | 3.95E-08 | -8.4993945 | 0.565 | 0.768 | 0.00092413 |
| ZNF667-AS1 | 3.96E-08 | -1.6769778 | 0.232 | 0.5 | 0.00092793 |
| ARPC2 | 4.82E-08 | -7.876377 | 0.75 | 0.878 | 0.00112861 |
| NME4 | 5.32E-08 | -1.6418372 | 0.576 | 0.78 | 0.0012444 |
| EGR1 | 5.34E-08 | -4.9570286 | 0.151 | 0.378 | 0.00124956 |
| NFIB | 5.62E-08 | -1.6611105 | 0.255 | 0.512 | 0.00131649 |
| SOX2 | 5.93E-08 | -5.2355441 | 0.016 | 0.122 | 0.00138826 |
| DOCK9 | 6.06E-08 | -1.1408411 | 0.108 | 0.317 | 0.00141755 |
| CNN3 | 6.18E-08 | -9.7227564 | 0.543 | 0.805 | 0.00144644 |
| KATNBL1 | 6.36E-08 | -0.5632155 | 0.229 | 0.5 | 0.00148891 |
| EHD4 | 6.84E-08 | -1.1391831 | 0.101 | 0.305 | 0.00160212 |
| RPS28 | 6.99E-08 | 145.697395 | 1 | 1 | 0.00163586 |
| DDX5 | 7.15E-08 | 2.86614819 | 0.704 | 0.866 | 0.0016728 |
| NR2F1 | 7.32E-08 | -1.7210366 | 0.029 | 0.159 | 0.00171342 |
| PRSS23 | 8.09E-08 | -11.204996 | 0.36 | 0.585 | 0.00189359 |
| HIGD1A | 8.17E-08 | -1.1788707 | 0.337 | 0.598 | 0.00191257 |
| MAP1S | 8.47E-08 | -1.1308101 | 0.087 | 0.28 | 0.0019839 |
| UBE2J1 | 8.88E-08 | -3.8645883 | 0.298 | 0.537 | 0.00207946 |
| TPBG | 9.04E-08 | -2.3109286 | 0.062 | 0.232 | 0.00211727 |
| HSPH1 | 9.25E-08 | -1.3434573 | 0.095 | 0.293 | 0.00216558 |
| EGLN1 | 9.74E-08 | -0.781441 | 0.108 | 0.317 | 0.00227933 |
| VEGFC | 9.93E-08 | -0.6020196 | 0.034 | 0.171 | 0.00232367 |
| RTN4 | 1.02E-07 | -1.8983065 | 0.563 | 0.756 | 0.00237856 |
| LIX1 | 1.06E-07 | -2.5762489 | 0.013 | 0.11 | 0.00247393 |
| APLP1 | 1.06E-07 | -1.051182 | 0.021 | 0.134 | 0.00248714 |
| FAM241A | 1.10E-07 | -1.4967345 | 0.097 | 0.293 | 0.00257547 |
| RPL38 | 1.11E-07 | 37.4279178 | 0.998 | 1 | 0.00259456 |
| SESTD1 | 1.12E-07 | -0.7493277 | 0.093 | 0.293 | 0.00261067 |
| HBG2 | 1.18E-07 | 525.841081 | 0.347 | 0.061 | 0.00276445 |
| SFRP2 | 1.26E-07 | -9.3303992 | 0.021 | 0.134 | 0.00294406 |
| ZMAT3 | 1.32E-07 | -0.8671575 | 0.159 | 0.39 | 0.0030852 |
| ITGA9 | 1.35E-07 | -3.4108011 | 0.157 | 0.39 | 0.00315862 |
| CD200 | 1.36E-07 | -2.4473847 | 0.154 | 0.378 | 0.00317568 |
| IPO11 | 1.41E-07 | -9.4777372 | 0.322 | 0.549 | 0.00329529 |
| TPM4 | 1.50E-07 | 3.81507386 | 0.694 | 0.817 | 0.00350491 |
| PEA15 | 1.55E-07 | -3.0825309 | 0.39 | 0.646 | 0.00363776 |
| SYT11 | 1.56E-07 | -0.3021266 | 0.021 | 0.134 | 0.00364673 |
| HNRNPA2B1 | 1.70E-07 | -11.549967 | 0.705 | 0.89 | 0.00398825 |
| ITFG1 | 1.74E-07 | -0.4537447 | 0.095 | 0.293 | 0.00408243 |
| DYNLL1 | 1.76E-07 | 3.1434999 | 0.885 | 0.951 | 0.00411929 |
| FAM89A | 1.89E-07 | -7.4423125 | 0.318 | 0.561 | 0.00442764 |
| HOXB5 | 1.96E-07 | -1.313381 | 0.126 | 0.341 | 0.00458819 |
| MSX1 | 2.05E-07 | -0.9493382 | 0.059 | 0.22 | 0.00480821 |
| YWHAQ | 2.08E-07 | -2.1226317 | 0.63 | 0.793 | 0.00486191 |
| TCF4 | 2.13E-07 | -16.746809 | 0.599 | 0.78 | 0.00497544 |
| SRSF3 | 2.15E-07 | 5.75493542 | 0.813 | 0.951 | 0.00502387 |
| PHPT1 | 2.23E-07 | 12.65853 | 0.658 | 0.817 | 0.00522111 |
| DOCK4 | 2.25E-07 | -0.6655658 | 0.119 | 0.329 | 0.00526247 |
| ANAPC13 | 2.26E-07 | -2.0575788 | 0.511 | 0.72 | 0.00529386 |
| ZSCAN9 | 2.31E-07 | -0.3603702 | 0.018 | 0.122 | 0.00539663 |
| CALM1 | 2.35E-07 | -10.248432 | 0.884 | 0.915 | 0.00550559 |
| RPAP2 | 2.36E-07 | -0.3370376 | 0.041 | 0.183 | 0.00551411 |
| PRND | 2.44E-07 | -8.6447678 | 0.162 | 0.378 | 0.00572062 |
| AC087627.1 | 2.63E-07 | -1.1801187 | 0.126 | 0.329 | 0.00616035 |
| MED17 | 2.66E-07 | -0.5745376 | 0.077 | 0.256 | 0.00622155 |
| CCND1 | 2.76E-07 | -5.3701488 | 0.155 | 0.378 | 0.0064707 |
| APOLD1 | 2.87E-07 | -4.0908221 | 0.126 | 0.329 | 0.00672054 |
| MAP4K4 | 2.91E-07 | -7.2771319 | 0.465 | 0.695 | 0.00682104 |
| YES1 | 2.98E-07 | -1.6663204 | 0.188 | 0.427 | 0.00697636 |
| SKAP1 | 3.53E-07 | -0.8855026 | 0.023 | 0.134 | 0.00827145 |
| AUTS2 | 3.67E-07 | -0.8566449 | 0.165 | 0.39 | 0.00860142 |
| CHMP3 | 3.80E-07 | -2.5201074 | 0.337 | 0.61 | 0.00888942 |
| GRK5 | 4.03E-07 | -1.2810616 | 0.155 | 0.366 | 0.00943457 |
| TSPO | 4.05E-07 | -0.6202526 | 0.028 | 0.146 | 0.00949313 |
| OTUD6B-AS1 | 4.10E-07 | -0.7317307 | 0.165 | 0.39 | 0.00959784 |
| MYO10 | 4.23E-07 | -3.4737828 | 0.203 | 0.439 | 0.00991009 |
| RAP2B | 4.26E-07 | -0.6074831 | 0.11 | 0.305 | 0.00997755 |
| MDM2 | 4.30E-07 | -0.9480727 | 0.095 | 0.28 | 0.01007564 |
| SUB1 | 4.44E-07 | 1.97438531 | 0.809 | 0.939 | 0.01039097 |
| TTC37 | 4.55E-07 | -0.5519568 | 0.079 | 0.256 | 0.01065923 |
| TGFBR2 | 4.58E-07 | -2.444238 | 0.152 | 0.366 | 0.01071222 |
| RPS26 | 4.73E-07 | -0.9509693 | 0.987 | 1 | 0.01107949 |
| FGFBP3 | 5.29E-07 | -2.6007798 | 0.044 | 0.183 | 0.01238012 |
| KLHL2 | 5.37E-07 | -0.4082641 | 0.049 | 0.195 | 0.01258083 |
| DNAJB6 | 5.59E-07 | -1.1797088 | 0.506 | 0.732 | 0.01308966 |
| SEC14L1 | 5.68E-07 | -4.0992499 | 0.268 | 0.476 | 0.01329578 |
| GPR4 | 5.80E-07 | -1.5768041 | 0.097 | 0.28 | 0.01357094 |
| CHD9 | 5.89E-07 | -1.5547993 | 0.239 | 0.476 | 0.01380063 |
| STX6 | 6.13E-07 | -0.8061616 | 0.082 | 0.256 | 0.01434102 |
| SVIP | 6.82E-07 | -1.8096168 | 0.264 | 0.512 | 0.01597738 |
| MYLK | 7.20E-07 | -0.3882982 | 0.044 | 0.183 | 0.01685837 |
| SF1 | 7.34E-07 | -0.8663459 | 0.406 | 0.695 | 0.01718564 |
| H2AFJ | 7.38E-07 | 2.72033859 | 0.489 | 0.22 | 0.01728628 |
| TMEM88 | 7.48E-07 | 34.576686 | 0.612 | 0.317 | 0.01751695 |
| TOP2B | 7.48E-07 | -0.8175833 | 0.144 | 0.354 | 0.0175227 |
| MFNG | 7.50E-07 | -0.6593484 | 0.025 | 0.134 | 0.01756942 |
| EID1 | 7.59E-07 | -6.6073023 | 0.71 | 0.841 | 0.01777729 |
| SBDS | 8.11E-07 | -1.0773668 | 0.172 | 0.39 | 0.01898903 |
| GIMAP4 | 8.34E-07 | -11.547474 | 0.206 | 0.439 | 0.01951672 |
| DSTN | 8.39E-07 | -5.6382965 | 0.684 | 0.866 | 0.01963858 |
| MRPS26 | 8.45E-07 | -1.3838356 | 0.27 | 0.512 | 0.01977442 |
| NIPA2 | 8.47E-07 | -1.0046483 | 0.203 | 0.439 | 0.01983278 |
| APC | 8.52E-07 | -0.4644919 | 0.075 | 0.244 | 0.0199474 |
| HES1 | 8.77E-07 | -23.713821 | 0.509 | 0.695 | 0.02054265 |
| RNF130 | 8.87E-07 | -0.9679692 | 0.19 | 0.415 | 0.02075611 |
| CARHSP1 | 9.80E-07 | -2.0747562 | 0.645 | 0.805 | 0.02293657 |
| SMAD1 | 1.00E-06 | -0.864137 | 0.129 | 0.329 | 0.02343756 |
| ATG4A | 1.01E-06 | -1.0481908 | 0.118 | 0.305 | 0.02360194 |
| ZFAS1 | 1.02E-06 | 18.5380762 | 0.933 | 0.78 | 0.0238903 |
| PLVAP | 1.05E-06 | 7.20136481 | 0.412 | 0.622 | 0.02451597 |
| MYO1B | 1.09E-06 | -1.3154725 | 0.133 | 0.329 | 0.02549881 |
| PLEKHA1 | 1.11E-06 | -1.308266 | 0.162 | 0.378 | 0.02605099 |
| ARRDC3 | 1.12E-06 | 0.39748006 | 0.272 | 0.524 | 0.02623106 |
| RNF165 | 1.12E-06 | -0.2767738 | 0.016 | 0.11 | 0.0263211 |
| FSCN1 | 1.13E-06 | -12.576997 | 0.678 | 0.793 | 0.02652877 |
| ESF1 | 1.17E-06 | -0.4247769 | 0.097 | 0.28 | 0.02730149 |
| ZFHX3 | 1.29E-06 | -1.1022073 | 0.118 | 0.305 | 0.03019508 |
| ITSN2 | 1.49E-06 | -1.0442877 | 0.118 | 0.305 | 0.03495155 |
| CD47 | 1.50E-06 | -0.8742494 | 0.227 | 0.463 | 0.035104 |
| CBL | 1.50E-06 | -0.6406124 | 0.085 | 0.256 | 0.03512525 |
| SLC9A3R2 | 1.55E-06 | -2.1961853 | 0.398 | 0.585 | 0.03625854 |
| KLHL7 | 1.58E-06 | -0.4672396 | 0.092 | 0.268 | 0.03704083 |
| ELAVL2 | 1.60E-06 | -0.3525207 | 0.021 | 0.122 | 0.03738039 |
| HAGLROS | 1.61E-06 | -0.3946192 | 0.021 | 0.122 | 0.03779574 |
| HTATSF1 | 1.66E-06 | -0.6322318 | 0.065 | 0.22 | 0.03893257 |
| DUSP6 | 1.72E-06 | -1.8609792 | 0.25 | 0.476 | 0.04015273 |
| ABCG2 | 1.72E-06 | -0.5993445 | 0.036 | 0.159 | 0.04038059 |
| RNF150 | 1.79E-06 | -0.2500242 | 0.021 | 0.122 | 0.04185958 |
| RGS5 | 1.99E-06 | -4.0799641 | 0.439 | 0.646 | 0.04665642 |
| KIF1BP | 2.00E-06 | -0.3010324 | 0.026 | 0.134 | 0.04680328 |
| PON2 | 2.01E-06 | -1.5728755 | 0.134 | 0.329 | 0.04700545 |
| HIST2H2BE | 2.01E-06 | -0.4143903 | 0.079 | 0.244 | 0.0470504 |
| RPS3A | 2.03E-06 | 21.6284076 | 0.998 | 1 | 0.04752039 |
| NUTM2A-AS1 | 2.05E-06 | -0.7205853 | 0.155 | 0.366 | 0.04808905 |


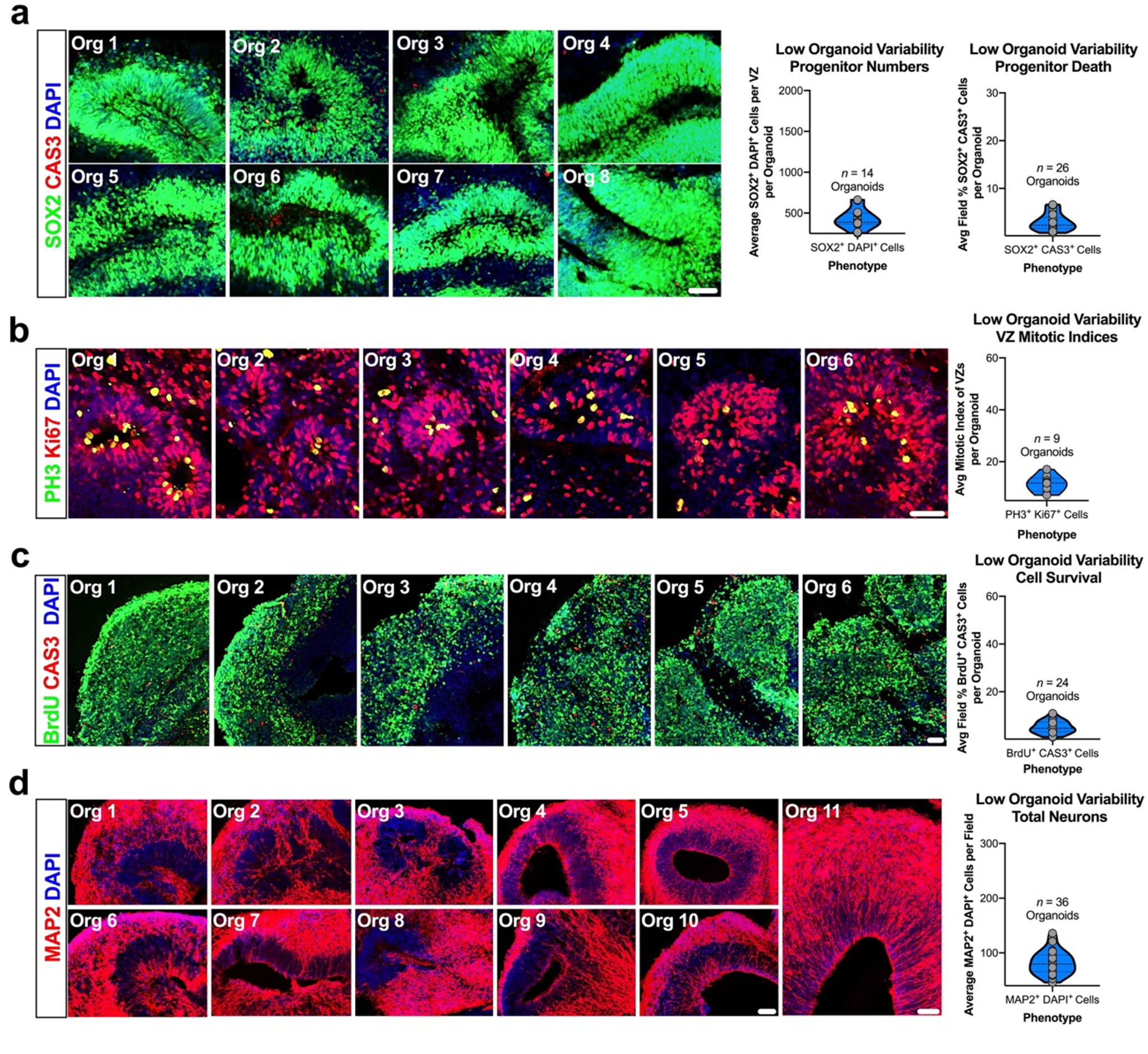


**Supplementary** **Figure 1: Variability of cerebral organoids generated using morphogen-free protocol.**

At the time we performed the scRNA-seq experiments for our previous study using the SCZ lines [13], we were restricted by the number of the patient lines that were available. Thus, initially, both for proteomics and scRNA-seq experiments, we used 3 lines per group. During the manuscript/publication phase of Notaras *et al*., *Molecular Psychiatry* 2022, we obtained additional CTRL and SCZ lines. We used the new patient lines (making up to 17) to empower the proteomics studies (also for a follow up paper: Notaras *et al*., *Translational Psychiatry* 2022- The proteomic architecture of schizophrenia iPSC-derived cerebral organoids reveals alterations in GWAS and neuronal development factors). This allowed us to unbiasedly monitor organoid reproducibility using 25 independent lines. Orthogonal LC/MS analysis split by iPSC line supported organoid reproducibility, revealed no major evidence of batch effects, and confirmed inter-group reproducibility (please see ref 13 in the manuscript).

For the current study, we used immunohistochemistry to measure variability of outcome measures that positively reflect organoid health and organization. We performed multiple non-overlapping quantifications in single organoids, generated an organoid average, and compared this average expression difference to determine variability between organoids. This high-content analysis resulted in hundreds of non-overlapping quantifications from ventricular zones and local fields, derived from a comparatively large number of organoids.

**a-c. Cerebral organoids exhibit low baseline variability in progenitor pools.** Analysis of multiple regions across n = 14 organoids revealed low variability in average progenitor numbers within the ventricular zones of cerebral organoids from healthy subjects. In an even larger analysis of n = 26 organoids, variability remained similarly low for progenitor survival between organoids. Cerebral organoids also exhibited reproducible ventricular zone mitotic activity across n = 9 individual organoids. To examine new-born cell survival, we adapted BrdU pulse-chase assay. Co-staining n = 24 individual organoids for BrdU and the cleaved, activated, form of Caspase-3 revealed low and reproducible rates of nascent cell death. Thus, despite differences in morphology, progenitor pool phenotypes were shown to exhibit reproducibility at baseline.
**d. Cerebral organoids exhibit reproducible enrichment of neurons**. To examine variance in neuron numbers within developing cortical fields, n = 36 organoids were stained for MAP2. As can be seen from inset images of cortical fields from 11 different organoids, a robust enrichment of neurons was readily apparent across organoids. When combined, these data established a baseline for cellular variability within cerebral organoids and confirmed that our organoid cultures exhibited suitably low variability for disease modeling within a morphogen-free 3D macroenvironment. Each dot on graphs represents the average of a single cerebral organoid. Scale: 60µm.

*.*

**
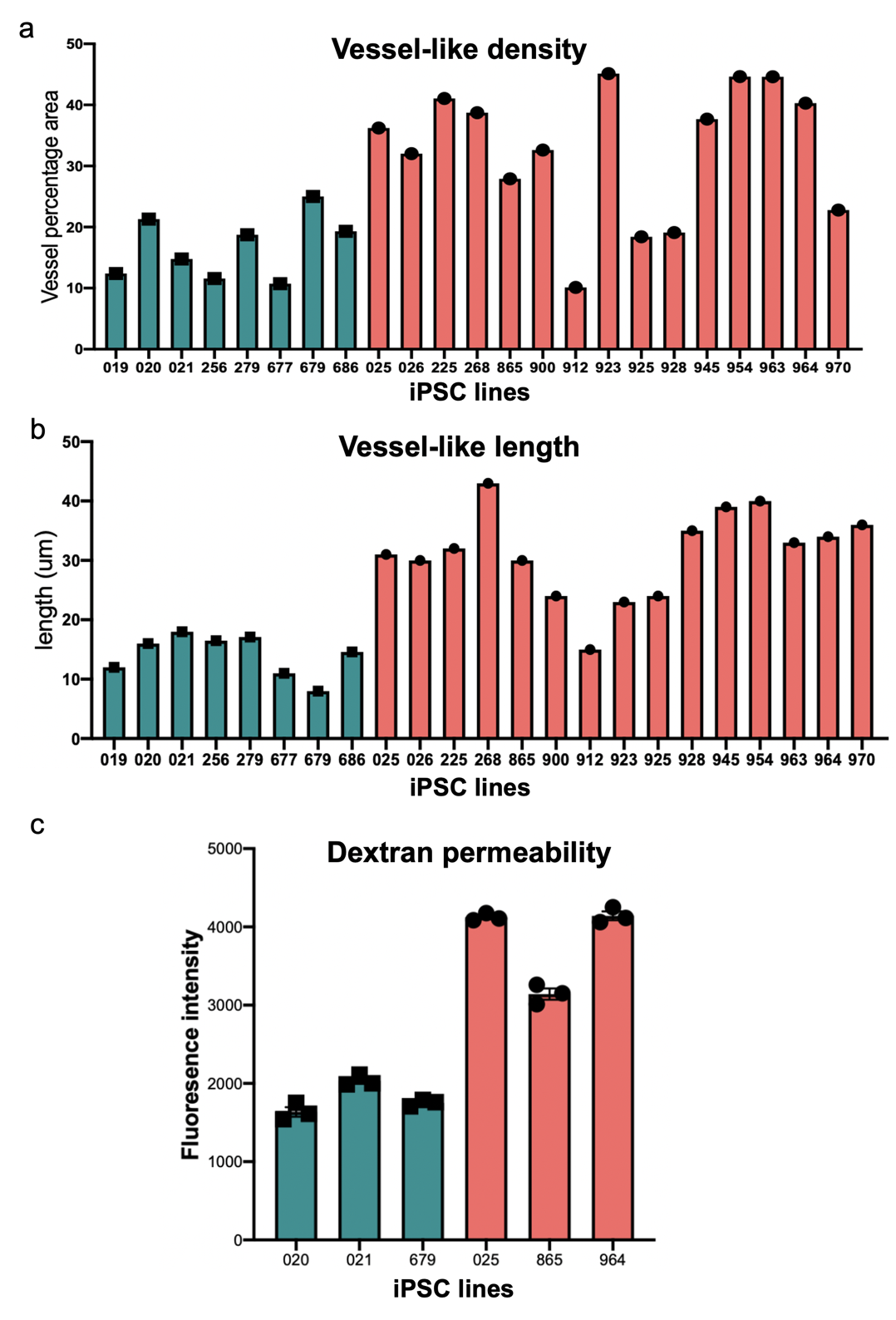
Supplementary** **Figure 2: Data split analyses by individual CTRL and SCZ lines for quantifications in Figure 1 and Figure 5.**

**a, b. PECAM+ vessel-like density as well as length phenotypes across lines.** For each donor line we assessed multiple organoids and measured multiple fields of view. When averaged, data from 15 SCZ lines exhibited higher PECAM+ densities compared to CTRL lines (**Figure 2e**). However, data split analysis by lines revealed that SCZ lines MH0185912, MH0185925, MH0185928, and MH0185970 were outliers compared to other SCZ lines in panel **a**. Similarly, we observed that the SCZ line MH0185912 exhibited microvascular-like vessel length that is comparable to the length of CTRLs in panel **b**. Together, an overall increase in microvascular-like vessel density and length together with low variability were observed for SCZ lines when compared to CTRLs. Each data point represents a mean of multiple fields of view (from at least 5 organoids) for a single line. Error bars reflect Standard Error of the Mean.

**c. 2D induced BMECs exhibit low variability across iPSC lines in dextran permeability assay.** After differentiation of CTRL or SCZ iPSC lines into BMECs, 3 technical replicates were used for each line (3 lines per group). See the details of the workflow of FITC-dextran assay to measure paracellular permeability of cultured iBMECs in **Figure 5**. SCZ iBMECs exhibited significantly higher permeability than CTRL lines (see the average in **Figure 5**). Graph depicts quantifications of FITC-dextran fluorescence permeability assay per line. Line split analysis depicts that fluorescent intensity did not show high variability across the lines for each group. Error bars reflect Standard Error of the Mean.
